# Three-Dimensional Reconstruction and Real-Time Deformation of Flexible Bodies: A Scoping Review (2009–2025)

**DOI:** 10.3390/s26134007

**Published:** 2026-06-24

**Authors:** Silvia Zisu, Silviu Butnariu

**Affiliations:** Department of Automotive and Transport Engineering, Transilvania University of Brasov, 29 Eroilor Blvd, RO-500036 Brasov, Romania; silvia.zisu@unitbv.ro

**Keywords:** 3D reconstruction, non-rigid tracking, flexible bodies, real-time deformation, RGB-D, LiDAR, tactile sensing, physics-based simulation, data-driven modeling

## Abstract

**Highlights:**

This scoping review synthesizes the current state of flexible body modeling research, identifying critical gaps and charting the most promising directions for future development.

**What are the main findings?**

- A scoping review of 56 peer-reviewed works (2009–2025) identifies four systemic gaps in flexible body modeling: absent physics-aware benchmarks, unresolved speed–accuracy trade-offs, fragile occlusion handling, and limited end-to-end sensing–simulation integration.
- Neural and implicit representations (20%), FEM-based methods (16%), and hybrid pipelines (21%) dominate recent contributions, with 60% of works published between 2022–2025.

**What are the implications of the main findings?**

- Model order reduction, differentiable physics, and multimodal sensing fusion are the most promising directions toward real-time, physically consistent digital twins of deformable environments.
- A coordinated community effort to build unified, physics-aware benchmarks is the most pressing need for advancing fair evaluation across reconstruction and simulation paradigms.

**Abstract:**

Following the PRISMA-ScR framework for scoping reviews, we systematically searched five databases (Scopus, IEEE Xplore, ScienceDirect, SpringerLink, Web of Science) using a Boolean query combining real-time processing, 3D reconstruction, and deformation modelling terms. From 86 records identified, 56 peer-reviewed publications (2009–2025) were retained after two-stage screening and organized into a unified taxonomy covering sensing modalities (RGB-D, LiDAR, tactile), reconstruction pipelines (volumetric fusion, NRSfM, neural radiance fields), and deformation models (FEM, PBD, mass-spring, GNN-based surrogates, differentiable simulators). Of the 56 included works, 60% were published between 2022 and 2025, confirming the field’s rapid growth. Neural and implicit representations account for 20% of contributions, FEM-based methods for 16%, and hybrid or application-specific pipelines for 21%. Four systemic gaps emerge: the absence of a unified physics-aware benchmark; unresolved speed–accuracy trade-offs (PBD achieves >30 FPS on desktop GPUs for 10^3^–10^4^ vertex meshes but lacks mapping to physical material constants (Young’s modulus, Poisson’s ratio), limiting material fidelity; full-order FEM ensures physically consistent stress–strain behavior but runs at only 1–10 FPS without order reduction; reduced-order FEM recovers interactive rates for low-frequency deformation modes); fragile handling of occlusions and multi-object contact; and limited end-to-end integration of sensing and simulation. The findings support the presentation of a research roadmap centered on model order reduction, differentiable physics, multimodal sensing fusion, and standardized evaluation protocols, with implications for robust digital twins of deformable environments.

## 1. Introduction

The modeling of flexible objects has gained significant momentum in recent years, propelled by tangible and urgent needs arising from a number of high-impact application domains. In soft robotics, robots must predict and respond to the deformation of grasped objects in real time—for instance, estimating how a silicone gripper deforms around a fragile component on an assembly line, or how a textile item reshapes during automated folding [1]. In intraoperative surgical simulation, non-rigid reconstruction of soft tissue is essential for updating pre-operative anatomical models to reflect breathing motion and tool-induced deformation during minimally invasive procedures, directly affecting the accuracy of tumor localization and instrument guidance [2]. In digital fashion and garment reconstruction, recovering the three-dimensional geometry of deforming clothing items from monocular video enables virtual try-on systems and physically accurate avatar animation [3]. Advances in sensing technologies and machine learning have enabled significant progress in capturing and reconstructing deformable objects across all these domains, yet the field remains fragmented, with no unified framework connecting sensing, reconstruction, and simulation into a coherent real-time pipeline.

Nevertheless, the body of existing work has developed in a largely disjointed fashion, with distinct research communities addressing sensing, geometric reconstruction, and physical simulation in isolation from one another. As a result, there is currently no unified perspective on how these components interact within real-time systems.

Most approaches treat reconstruction and deformation as loosely coupled stages, which leads to inconsistencies in geometry, instability in simulation, and reduced robustness under occlusions or complex interactions. In addition, the rapid emergence of neural representations and differentiable simulation has introduced new paradigms that remain insufficiently integrated into the broader landscape. Therefore, a comprehensive review systematically organizes sensing modalities, reconstruction pipelines, and deformation models—while explicitly addressing their integration and limitations—is both timely and necessary. Such a synthesis is essential for advancing toward robust, real-time systems capable of modeling complex deformable environments.

This review surveys the state of the art in 3D reconstruction and real-time deformation of flexible bodies from 2009 to 2025, with a focus on methods that operate at near interactive rates. We structure the discussion around three core building blocks: sensing modalities (RGB-D, stereo, LiDAR, and tactile systems), reconstruction pipelines for static and dynamic geometry, and deformation models ranging from classical finite element and mass-spring systems to position-based dynamics and data-driven, neural approaches. Within each block, we highlight representative algorithms, their assumptions, and their computational profiles, emphasizing how they address occlusions, large deformations, multi-object scenes, and contact-rich interactions.

This review makes two distinct contributions. On one hand, it brings together a rapidly expanding and increasingly diverse body of work under a unified taxonomy—one that meaningfully connects decisions around sensing, reconstruction, and simulation to their real-world consequences for the manipulation of flexible bodies. On the other hand, it lays out a concrete roadmap for future research, advocating for standardized, physics-aware benchmarks alongside hybrid algorithms that draw on both model-based and data-driven approaches, perception systems capable of handling occlusion and contact, and learning frameworks enriched by differentiable physics.

Taken together, these directions are intended to serve both those entering the field and seasoned researchers alike, guiding collective progress toward digital twins of deformable environments that are at once robust and capable of operating in real time.

## 2. Methodology

This work follows a scoping-review design to systematically map the landscape of 3D reconstruction and real-time deformation methods for flexible bodies, rather than to perform a quantitative meta-analysis. The goal is to capture the breadth of sensing modalities, reconstruction pipelines, and deformation models used between 2009 and 2025, to organize them into a coherent taxonomy, and to identify persistent gaps and emerging directions.

The review is informed by the PRISMA-ScR (Preferred Reporting Items for Systematic Reviews and Meta-Analyses extension for Scoping Reviews) framework and adapts its key steps to the conventions of computer vision, robotics, and computer graphics.

### 2.1. Eligibility Criteria and Study Selection

Studies were included if they constituted original research articles published in peer-reviewed journals, were written in English, were available in full text, and had been published after 2009. Studies were excluded when they consisted of commentaries, editorials, short communications, magazine articles, dissertations, book chapters, other non-academic publications, or when full-text access was not attainable. The selection was limited to journal articles and conference papers with a peer review process to ensure a high level of scientific rigor. Screening was performed in two stages: first, titles and abstracts were examined against the inclusion and exclusion criteria; second, the remaining full-text articles were assessed to confirm eligibility.

**Figure 1:**
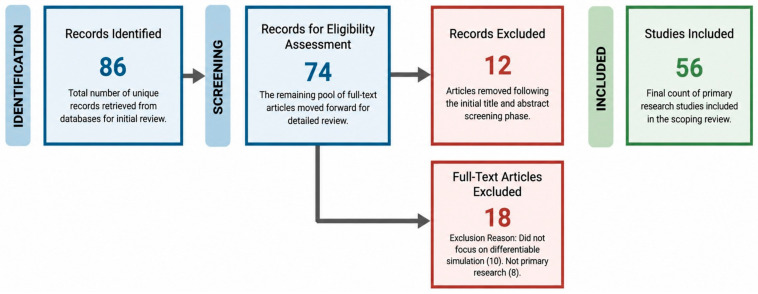
PRISMA diagram.

In accordance with PRISMA-ScR recommendations, the two-stage screening process was conducted independently by both authors. In the first stage, the titles and abstracts of all 86 retrieved records were screened separately by S.Z. and S.B. against the predefined inclusion and exclusion criteria. Disagreements were resolved through structured discussion until consensus was reached; no third-party arbitration was required. Inter-rater agreement at this stage was assessed using Cohen’s kappa coefficient, yielding κ = 0.81, which corresponds to almost perfect agreement according to the Landis and Koch scale. In the second stage, the full texts of the 74 remaining records were assessed independently by both authors. Disagreements at this stage concerned primarily the categorization of borderline conference papers and papers with partial overlapping with the review scope; all were resolved by consensus discussion. The final inclusion of 56 publications was agreed upon by both authors without unresolved conflicts. This dual-reviewer process ensures that the selection decisions reported in the PRISMA diagram (Figure 1) are reproducible and free from single-reviewer bias.

Inclusion criteria: Studies were included if they satisfied all of the following conditions: (i) original research articles or full conference papers published in peer-reviewed venues; (ii) written in English; (iii) available in full text through institutional access; (iv) published between January 2009 and March 2025; (v) addressing at least one of the three core components of the review scope—sensing modalities for deformable objects, 3D reconstruction pipelines for non-rigid scenes, or real-time deformation modeling—and doing so in the context of flexible or deformable bodies as defined in Section 1.

Exclusion criteria: Studies were excluded if they met any of the following conditions: (i) commentaries, editorials, letters, short communications, magazine articles, or opinion pieces; (ii) master’s theses, doctoral dissertations, or technical reports not accompanied by a peer-reviewed publication; (iii) book chapters or monographs; (iv) full text not accessible; (v) primary focus on rigid-body reconstruction or simulation without deformable components; (vi) purely theoretical contributions without computational implementation or empirical evaluation; (vii) redundant publications presenting results already covered by a higher-quality or more comprehensive work already included in the corpus, assessed on the basis of overlapping datasets, methods, and results sections.

Borderline cases: Records that could not be unambiguously classified against the above criteria—primarily conference papers with partial thematic overlap and extended abstracts of journal papers—were flagged during Stage 1 screening and resolved by consensus discussion between both authors during Stage 2 full-text assessment.

The PRISMA diagram is shown in Figure 1. The synthetic summary table of the included works for the scoping review, grouped by methodology is presented in Appendix A.

**Figure 2:**
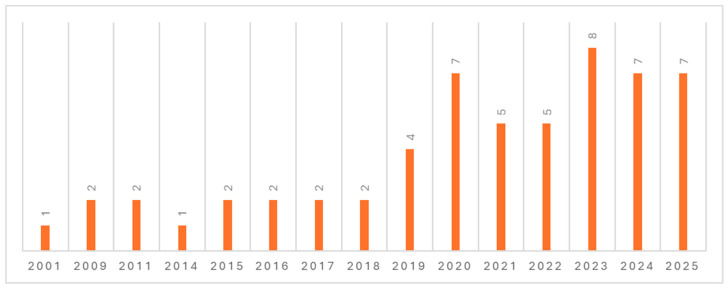
Number of research papers during the period studied.

Regarding the chronology of articles (Figure 2): the analysis shows a pronounced concentration of research in recent years, with 33 articles (approximately 60% of the total library) published in the period 2022–2025 alone. This pattern is consistent with the field of differentiable physical simulation and non-rigid reconstruction being an area of active and growing interest, reflecting a sharp increase in publication volume over the last four years.

**Figure 3:**
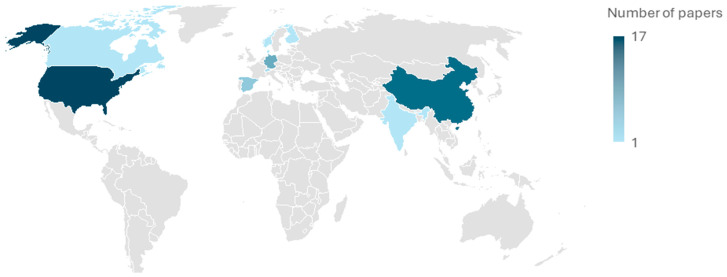
Research papers repartition on the world.

Based on manual verification of author affiliations in the content and metadata of the 56 articles studied, we identified the country of origin of the first author for each paper. The results, shown in Figure 3, reflect a global distribution, with a clear dominance of research institutions in the United States (especially MIT and Stanford) and China (Zhejiang University, Shanghai Jiao Tong), with the following observations: United States (17 articles)—represents the main research hub, with a strong focus on differentiable simulations and soft robotics (MIT/Stanford); China (13 articles)—has a strong presence in the fields of 3D reconstruction and tactile sensors; Europe (20 articles)—when viewed as a bloc, Europe is highly competitive, with Germany (7) and the United Kingdom (5) leading efforts in graph-based simulations and computer vision.

**Figure 4:**
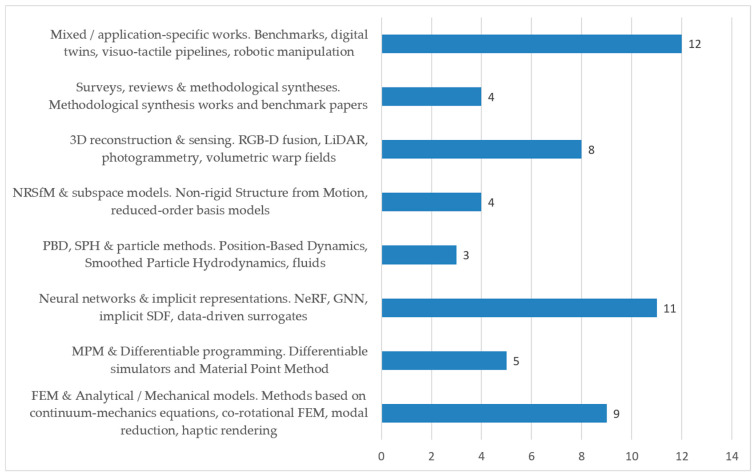
Distribution of research papers according to the topic studied.

International affiliations: Many papers are the result of collaborations between multiple countries (e.g., Spain–France or USA–China collaborations), but the table above prioritizes the affiliations of the first author as requested. Figure 4 graphically presents the number of papers divided by the fields studied.

### 2.2. Data Extraction and Quality Assessment

For each of the 56 included works, data was extracted systematically using a standardized extraction form developed prior to screening. The form captured the following fields: (i) bibliographic metadata (authors, year, venue, country of first author affiliation); (ii) sensing modality or modalities employed; (iii) reconstruction pipeline category, mapped to the taxonomy presented below; (iv) deformation model category, mapped to the same taxonomy; (v) application domain; (vi) whether the method operates in real time (defined operationally as ≥25 FPS on the hardware reported by the authors); (vii) evaluation datasets and metrics reported; and (viii) key quantitative results where available (FPS, Chamfer Distance, or equivalent). Extraction was performed by the first author (S.Z.) and verified for a random 30% sample by the second author (S.B.); discrepancies were resolved by returning to the original source.

We adopted a conservative threshold of 25 FPS to ensure perceptual smoothness in interactive graphics, consistent with prior real-time reconstruction surveys. This baseline is intentionally lower than the standard 30 FPS to accommodate borderline systems described as real-time by their creators. However, readers should note that this uniform threshold covers highly heterogeneous hardware, from embedded platforms to high-end GPUs. A reported 25 FPS simply indicates real-time performance on the authors’ specific configuration, rather than universal hardware compatibility. We address these hardware-related limitations in detail in Section 9.1.

As a scoping review, this work does not apply formal quality appraisal instruments such as the Mixed Methods Appraisal Tool (MMAT) or the Newcastle–Ottawa Scale, which are designed for systematic reviews performing quantitative evidence synthesis. Instead, quality was assessed implicitly through the eligibility criteria: restriction to peer-reviewed venues, exclusion of grey literature, and exclusion of redundant or lower-quality duplicates (criterion vii above). This approach is consistent with PRISMA-ScR guidance, which explicitly states that formal quality appraisal is optional for scoping reviews and should be adapted to the review’s purpose. The absence of formal quality scores is acknowledged as a limitation in Section 2.4.

### 2.3. Review Questions and Scope

The review is guided by the following research questions:

RQ1: Which sensing modalities (e.g., RGB-D, stereo, LiDAR, tactile) are most effective for capturing deformable objects in real time, and under what conditions?

RQ2: How do existing 3D reconstruction pipelines handle non-rigid motion, occlusions, and multi-object scenes involving flexible bodies?

RQ3: What real-time deformation models are used for flexible bodies (e.g., FEM, mass-spring, PBD, neural surrogates), and what are their main computational and accuracy trade-offs?

RQ4: How are reconstruction and simulation integrated into end-to-end pipelines, and what are the primary limitations and open challenges?

The population of interest comprises methods that explicitly address deformable or flexible objects (cloth, soft tissue, cables, elastomers, soft robots, deformable tools or workpieces). The concept focuses on 3D reconstruction and/or real-time deformation simulation. The context covers applications in robotics, AR/VR, medical simulation, digital twins, and related interactive systems.

### 2.4. Limitations of This Review

Like any scoping review, this work comes with several limitations worth acknowledging. To begin with, the literature search was restricted to five specific databases and to publications written in English—a choice that may have inadvertently excluded relevant studies published in other languages or indexed through different channels. Beyond that, drawing on both journal articles and conference papers introduces a certain degree of methodological unevenness, since the two publication formats are held to different standards of peer review.

Furthermore, while the screening process incorporated independent verification steps, the review was conducted within a single research group, which may introduce a degree of selection bias not fully captured by the inter-rater agreement measure reported in Section 2.1.

The reliance on a specific set of keywords (FEM, mass-spring, PBD) and the rapid growth of the field—where 60% of the literature emerged between 2022 and 2025—suggests that some very recent or alternatively labeled studies might be missing. Finally, as a scoping review, this work does not include a formal quality appraisal or a quantitative meta-analysis of the data.

Finally, while data extraction followed a pre-specified form and was partially verified by a second reviewer, the absence of a formal quality appraisal instrument means that included works are treated as broadly equivalent in terms of methodological rigor, which may obscure differences in the reliability of reported quantitative results.

## 3. Sensing Modalities

### 3.1. Overview of Sensing Technologies

Accurate reconstruction of deforming objects in real time requires advanced technologies capable of capturing both geometry and motion. A variety of sensing modalities can be used for this purpose. RGB-D cameras represent one of the most versatile options, providing synchronized color (RGB) images and depth maps. These sensors—exemplified by devices like the Microsoft Kinect and Intel RealSense—project structured light or use time-of-flight to obtain depth measurements. Modern RGB-D systems operate at frame rates suitable for real-time applications [4] (often around 30 Hz), enabling dynamic scene capture. Stereo vision systems, using two or more calibrated cameras to infer depth via triangulation, offer higher potential resolution and range but require more complex calibration and are sensitive to texture for reliable matching. Specialized active methods include structured-light projectors and LiDAR scanners, each with different trade-offs between speed, accuracy, and operational range. Meanwhile, tactile sensing provides another layer of information: in robotics contexts, contact-based deformation measurements from soft tactile sensors can complement visual data, capturing force and deformation directly where the robot touches an object. Recent tactile devices even incorporate embedded depth sensors, producing rich signals that combine geometric shape sensing with force detection [1].

### 3.2. RGB-D/LiDAR

LiDAR (Light Detection and Ranging) is another modern sensing approach, which uses scanning laser beams to directly measure distances to surfaces. Spinning LiDAR systems, such as those used in autonomous vehicle platforms, typically achieve range accuracies of ±2–3 cm at distances up to 100 m and produce point clouds at 10–20 Hz, making them well suited for large-scale outdoor mapping [5]. Single-photon LiDAR systems achieve sub-centimeter range precision at longer distances but at lower spatial density per frame and require advanced filtering to suppress background noise [5]. In the context of flexible-body reconstruction, both variants are constrained by their relatively low temporal resolution compared to RGB-D cameras operating at 30 Hz, limiting their applicability to slowly deform objects or static scene components. It is widely used in autonomous vehicles and mapping. The cost and size of LiDAR sensors have historically been high, and while they produce accurate geometry, the data is sparse in time. Merging LiDAR with other modalities can yield both accuracy and completeness. For instance, some real-time systems fuse LiDAR point clouds with camera imagery to colorize the points or fill gaps. New single-photon LiDAR techniques even allow for photon-level sensitivity and have been combined with advanced filtering to achieve reconstruction in challenging scenarios. Despite significant advancements, LiDAR remains relatively costly and energy-intensive compared to passive or low-power sensing alternatives [5].

### 3.3. Tactile Sensing

Tactile sensing constitutes a fourth, complementary modality that is particularly valuable when visual data is partially or fully occluded—a frequent condition during contact-rich manipulation.

Unlike RGB-D cameras or LiDAR, which capture scene geometry remotely, tactile sensors measure contact forces and surface deformation directly at the point of interaction.

In robotics applications, soft tactile pads integrated into gripper fingers can estimate the local shape of a grasped flexible body, providing ground-truth constraints that vision cannot supply under occlusion.

Recent designs embed miniaturized depth cameras inside a silicone membrane, enabling simultaneous measurement of contact geometry and normal-force distribution at sub-millimeter resolution [2].

When fused with visual reconstruction pipelines, these visuo-tactile signals allow the system to regularize mesh deformation in occluded regions and prevent physically implausible mesh collapse.

A key limitation is sensor coverage: current tactile arrays are confined to small contact patches, so global shape estimation still depends primarily on visual modalities.

Extending tactile coverage—through stretchable multi-cell arrays or distributed proprioceptive skins—is an active area of development and a recurring theme in the benchmark and evaluation literature reviewed in Section 7 and Section 8.

Having surveyed the four principal sensing modalities—RGB-D, LiDAR, photogrammetry, and tactile sensing—we now turn to the reconstruction pipelines that process their output.

The following section examines how raw sensor data is transformed into three-dimensional geometric representations of flexible bodies, covering both classical and learning-based approaches.

## 4. Three-Dimensional Reconstruction Pipelines

Three-dimensional reconstruction is a rapidly evolving field that transforms two-dimensional sensor data into three-dimensional models, enabling applications in robotics, virtual reality, cultural heritage, and environmental monitoring. Approaches to 3D reconstruction can be broadly categorized into traditional methods, which rely on classical geometric principles, and modern methods, which leverage advanced sensors and machine learning.

### 4.1. Traditional Methods

Traditional methods of 3D reconstruction include stereo vision, structure-from-motion (SfM), and multi-view stereo (MVS) (Table 1). Stereo vision is based on the principle of binocular inequality: by identifying corresponding points in images captured from two or more cameras at different viewpoints, one can triangulate the 3D position of those points. Stereo vision has been widely used due to its relatively low hardware cost and passive sensing (just cameras). It is effective for well-textured scenes where matching between images is reliable. However, stereo struggles with texture-less surfaces and with significant occlusions (where parts of the scene visible using one camera are hidden from another).

Photogrammetry can integrate data from various sensors; for example, drones might capture images and record GPS/IMU data to aid reconstruction. Lighting variations can hinder algorithm performance, and processing large image datasets is often time-consuming, typically requiring hours rather than enabling real-time results [6].

Structure-from-Motion (SfM) generalizes the idea of stereovision to large collections of images taken from different viewpoints, without requiring a calibrated multi-camera setup. SfM algorithms simultaneously estimate the camera poses and a sparse set of 3D points by matching features across many images. This technique has been particularly useful for offline reconstructions from unordered image collections. SfM does not require pre-existing knowledge of camera intrinsics or extrinsic (though it can use them if available), making it very flexible.

Its downsides include heavy computational load (as the number of image pairs to match grows) and sensitivity to the quality of features: if images lack overlap or have motion blur, SfM may fail or produce fragmented results. Multi-view stereo (MVS) builds on the camera pose estimates from methods like SfM (or from known camera rig geometry) to produce dense 3D reconstructions. Given a set of images with known poses, MVS algorithms seek to recover dense geometry (often as point clouds or depth maps for each view) by combining information from multiple views of each surface point. Traditional MVS methods use pixel-wise similarity measures (such as normalized cross-correlation) and use geometric consistency constraints to iteratively refine a dense point cloud or mesh. MVS can achieve high-detail reconstructions for objects and scenes and has been used in applications like digital cultural heritage (scanning historical artifacts or sites) and accident scene reconstruction. However, it is computationally intensive, and like stereo, it requires good texture and lighting consistency. Recent improvements incorporate learning-based depth estimation to handle low-texture regions better, but challenges remain in scaling MVS to real-time [7].

### 4.2. Modern Methods

Modern methods of 3D reconstruction include the use of depth cameras, LiDAR, and photogrammetry. Depth cameras, such as those from Microsoft Kinect and Intel RealSense, capture both color and depth information, often using structured light or time-of-flight technology. They provide real-time depth maps, enabling fast 3D reconstruction of dynamic scenes. This makes them ideal for applications like gaming and gesture recognition. However, they have limited range (typically up to 5 m) and are sensitive to lighting conditions [8].

#### 4.2.1. Non-Rigid

Non-rigid 3D reconstruction has evolved from early volumetric fusion methods to sophisticated unsupervised deep learning frameworks (Table 2). A significant methodological advance was introduced by DynamicFusion, the first system to achieve dense, real-time, template-free non-rigid reconstruction [9,10]. It utilizes a 6D volumetric warp field to fuse observations into a canonical frame [9]. However, its depth-only approach is limited in tracking tangential motion and handling topological changes where surfaces move from closed to open [9,11].

Before presenting the individual algorithms, it is important to draw a clear distinction between the two fundamental paradigms addressed in this section: Non-Rigid Structure from Motion (NRSfM) and Shape-from-Template (SfT). Although both aim to recover the 3D geometry of deforming objects, they operate under fundamentally different assumptions and input requirements.

NRSfM is a template-free approach: it simultaneously estimates 3D shape and camera motion from a sequence of 2D observations (point trajectories or optical flow), without requiring any prior knowledge of the object’s geometry. Its feasibility relies on low-rank or sparsity-based priors over the space of deformations, which constrain the ill-posed nature of the problem. NRSfM is therefore well suited to scenarios where no reference model of the object is available, such as in the analysis of general, previously unseen deformable surfaces.

SfT is a template-dependent approach: it requires a known reference shape (the template) as a prerequisite and recovers deformation by fitting this template to new 2D or 3D observations. Because the template encodes strong geometric prior information, SfT can achieve more accurate and stable reconstructions under occlusion and sparse observations, but it cannot be applied when the object’s rest shape is unknown or when the object undergoes topological changes that invalidate the template.

In practice, the choice between NRSfM and SfT is dictated by the availability of prior geometric information: NRSfM is preferred for exploratory or general-purpose reconstruction pipelines, whereas SfT is the method of choice in controlled settings such as surgical simulation or cloth tracking, where a reference model can be acquired in advance.

This distinction will be revisited in Section 7.2 in the context of benchmark evaluation.

Addressing these limitations, VolumeDeform incorporates sparse RGB (SIFT) features as global anchor points alongside dense depth constraints [11]. This hybrid strategy significantly improves tracking robustness against drift and enables the system to handle scenes with low geometric variation that would otherwise fail with depth-only methods [11].

Moving away from explicit data association, KillingFusion introduces a correspondence-free variational approach [10]. By evolving level sets on Signed Distance Fields (SDFs) and enforcing a Killing motion constraint to preserve local isometry, it intrinsically handles complex topological changes, such as surfaces merging or splitting, which typically cause artifacts in mesh-based trackers [10].

Parallel to volumetric methods, Deep NRSfM pioneered an unsupervised deep learning approach using hierarchical sparse coding [12]. By reinterpreting the problem as a Deep Neural Network autoencoder, it can reconstruct 3D structures from 2D landmarks at category-scale. It is particularly effective at handling missing data from occlusions and weak perspective projections [12]. Building on this, Multi-view Multi-body NRSfM extends these capabilities to group-level pose estimation [13]. It uses dictionary learning to simultaneously estimate multiple skeletons and camera parameters from uncalibrated multi-view setups.

#### 4.2.2. Neural/Implicit Representation

The foundational study is NeRF [14], which represents static 3D scenes as continuous 5D neural radiance fields. It optimizes an MLP to map spatial coordinates and viewing directions to volume density and emitted radiance, achieving photorealistic results for complex geometry [14]. However, NeRF is limited to static environments [15].

The subsequent studies extend this framework to dynamic scenes (Table 3). D-NeRF introduces a methodology for non-rigid geometries by decomposing the problem into a canonical scene and a time-dependent deformation field. This allows synthesis from a sparse set of monocular views without requiring 3D ground truth [15]. Similarly, Li et al. present Neural Scene Flow Fields, which synthesize views across both space and time. Their unique contribution is the simultaneous optimization of separate static and dynamic MLPs, combined with a warping-based temporal loss to handle deformations in monocular videos [16].

Finally, Nerfies specializes in uncontrolled captures, such as mobile phone selfies. It improves upon previous deformation models by utilizing an SE(3) transformation field, which is more efficient than simple translation fields for encoding rotations. It also introduces a coarse-to-fine regularization strategy to prevent the model from getting stuck in local minima during the optimization of high-frequency details [17].

Unlike D-NeRF or the method by Li et al., Nerfies is specifically engineered to handle the variability inherent in informal human capture. Its use of SE(3) fields is a significant mathematical improvement, as it parameterizes complex rotations with fewer parameters than translation-based models, leading to better convergence. Furthermore, its coarse-to-fine regularization [17] provides a more robust solution to local-minima convergence issues common in dynamic neural optimization contexts, yielding improved generalization on unconstrained capture data.

### 4.3. Comparative Overview

Each 3D reconstruction method has its strengths and limitations. Stereo vision offers a low-cost setup but is confined to textured surfaces and relatively short-baseline scenarios. SfM can work with arbitrary image sets and discover both structure and motion, but it is computationally heavy and depends on robust feature detection. Depth cameras provide real-time, dense measurements that make dynamic scene scanning possible, though within a limited range and under certain lighting conditions. LiDAR gives unparalleled precision and range for static scenes, at the expense of cost and data volume. Photogrammetry provides high-detail reconstructions and flexibility in data sources but usually operates offline and requires careful capture conditions.

Recent trends in 3D reconstruction include hybrid sensor systems that combine multiple modalities to capitalize on their complementary advantages. For example, a system might use an RGB-D camera for core geometry and a set of auxiliary RGB cameras for capturing fine texture or filling in occluded viewpoints. Another trend is pushing real-time processing capabilities: with help of GPUs and dedicated processors, algorithms that once ran offline (like bundle adjustment in SfM or depth fusion in MVS) are being sped up dramatically. There have been reports of reconstructing large scenes at near real-time rates by distributing computation to GPUs. Machine learning has also entered this space: depth estimation from a single image using CNNs can serve as an additional input to fusion algorithms, and learning-based point cloud denoisers can improve the quality of raw sensor data on the fly [7].

The applications of 3D reconstruction span diverse fields—including robotics, medicine, and cultural heritage, yet the future of the discipline lies in the tighter integration of AI and neural implicit representations (such as NeRFs) to handle complex, real-time scenarios more efficiently. While current methods enable critical tasks like surgical assistance and environmental monitoring, emerging trends focus on transitioning offline processes to incremental systems and utilizing event cameras to capture high-speed motions without blur, further enhancing the fidelity of dynamic scene reconstruction [18,19].

Having reviewed the major 3D reconstruction pipelines—from classical stereo and SfM to modern NeRF-based and event-camera approaches—we now turn to the deformation models that operate on the recovered geometry.

The following section examines how reconstructed flexible bodies can be animated and simulated in real time, covering physics-based, data-driven, and hybrid strategies.

## 5. Real-Time Deformation Methods

**Figure 5:**
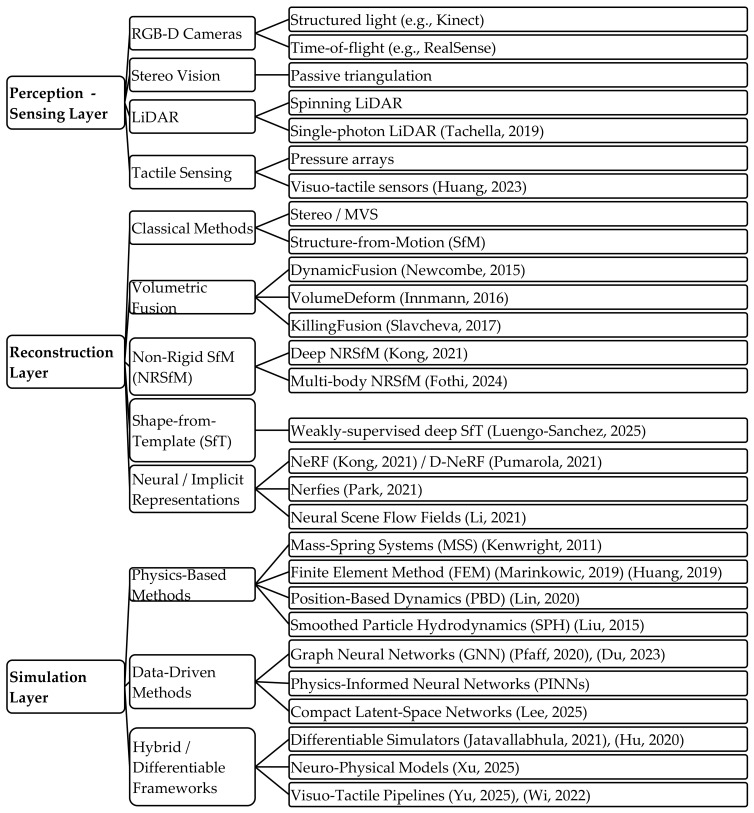
Taxonomy of 3D reconstruction and deformation for flexible bodies. Correspondence of bibliographical references: (Tachella, 2019)—[5], (Huang, 2023)—[1], (Newcombe, 2015)—[9], (Innmann, 2016)—[11], (Slavcheva, 2017)—[10], (Kong, 2021)—[12], (Fothi, 2024)—[13], (Luengo-Sanchez, 2025)—[27], (Pumarola, 2021)—[15], (Park, 2021)—[17], (Li, 2021)—[16], (Kenwright, 2011)—[28], (Marinkowic, 2019)—[20], (Huang, 2019)—[21], (Lin, 2020)—[24], (Liu, 2015)—[22], (Pfaff, 2020)—[21], (Du, 2023)—[29], (Lee, 2025)—[3], (Jatavallabhula, 2021)—[26], (Hu, 2020)—[30], (Xu, 2025)—[31], (Yu, 2025)—[32], (Wi, 2022)—[33].

Real-time deformation methods are crucial in fields including computer graphics, robotics, and engineering, where simulating the dynamic behavior of objects under external forces is essential. These methods enable the creation of realistic models that can interact with their environment in a physically plausible manner. Generally, two primary approaches have emerged for real-time deformation simulation: physics-based methods, which rely on explicit mathematical models of physical laws, and data-driven methods, which leverage machine learning to predict deformations from examples. This section provides an in-depth review of these approaches, highlighting their strengths, limitations, and applications. The three-layer structure of Figure 5 reflects a deliberate separation of concerns that guides the organization of this review. Sensing (Layer 1, covered in Section 3), reconstruction (Layer 2, covered in Section 4), and simulation (Layer 3, covered in Section 5) are treated as functionally distinct stages, each with its own assumptions, performance metrics, and failure modes. This separation does not imply that the three layers operate independently in practice—on the contrary, one of the central findings of this review is that their loose coupling is a primary source of system-level fragility (Section 8). However, analytical clarity requires that each layer be characterized on its own terms before their interactions are examined. Hybrid and differentiable frameworks, which explicitly bridge layers 2 and 3, are discussed both within Layer 3 (Section 5.5) and in the integrative analysis of Section 9.

On the computational side, flexible-body dynamics is supported by a wide range of modeling and simulation frameworks. The Finite Element Method (FEM) is widely regarded as the reference approach for accuracy in deformation simulation of solid elastic bodies, owing to its rigorous grounding in continuum mechanics and its ability to model both linear and nonlinear material behavior [20,21]. However, this characterization requires qualification: FEM accuracy is contingent on mesh quality, element type, and constitutive model selection, and it degrades significantly for problems involving topological changes such as tearing or cutting, large-deformation flows, or granular and fluid-like materials, where mesh-free methods such as SPH [22] or MPM [23] are more appropriate. The claim that FEM is the gold standard therefore applies specifically to elastic solid deformation under moderate strain, which is the primary scenario addressed in this review.

Traditionally, high-fidelity FEM was too slow for real-time use, but advances such as GPU acceleration and model order reduction have made real-time FEM increasingly feasible. By reducing a model’s degrees of freedom (e.g., via modal analysis) and leveraging parallel computation, FEM simulations can achieve interactive rates for moderate complexity, as detailed in studies of real-time FEM techniques [4].

Among the method families reviewed, geometric tracking methods and PBD-based simulators most consistently achieve frame rates above 30 FPS on standard desktop GPU hardware (NVIDIA RTX class) for objects of moderate mesh complexity (10^3^–10^4^ vertices) [21,24]. Compact latent-space neural networks, such as the cloth deformation model of Lee et al. [3], also achieve >100 FPS for specific object categories after offline training. Reduced-order FEM formulations with precomputed modal bases can reach 60+ FPS for low-dimensional deformation spaces [21,25], though this rate degrades rapidly as the number of retained modes or the complexity of contact scenarios increases. Full-order FEM and differentiable simulators consistently operate below real-time rates for the same hardware and mesh resolutions [20,26].

The taxonomy presented in Figure 5 is organized along the sequential stages of a reconstruction–simulation pipeline: Layer 1 (Sensing) covers the input modalities used to acquire geometric and contact information; Layer 2 (Reconstruction) covers the pipelines that transform raw sensor data into three-dimensional geometric representations; Layer 3 (Simulation) covers the deformation and dynamics models that operate on the recovered geometry. This separation reflects the functional architecture of integrated systems and enables independent assessment of design choices at each stage.

Physics-Based Methods (FEM, mass-spring, PBD, SPH) are defined as those whose dynamic behavior is derived analytically from continuum mechanics or Newtonian principles, independently of any training data. Data-Driven Methods, by contrast, are defined as those whose parameters—and, in some cases, whose functional form—are optimized from observed data rather than derived from first principles. Under this criterion, Neural Radiance Fields (NeRF) are classified within the Data-Driven category: a NeRF model has no a priori knowledge of scene geometry or deformation physics; its volumetric density and radiance functions are learned entirely through gradient-based optimization over a set of 2D observations.

While NeRF employs volumetric rendering equations that are physically inspired, these equations serve as a differentiable image-formation model rather than as a physics simulator of material behavior, and the model’s representational content is entirely data-derived. This distinguishes NeRF from physics-based methods, which enforce constitutive laws and conservation principles regardless of the observed data. It is acknowledged that NeRF-based representations occupy a boundary position within this taxonomy—closer to implicit geometric representations than to surrogate dynamics models such as GNN-based simulators—and this nuance is reflected in the dedicated subsection (Section 4.2.2) where NeRF methods are discussed separately from purely data-driven dynamics surrogates.

Data-Driven methods require significant GPU resources in the training phase but are extremely fast in execution (Inference).

From point of view of Robustness to Occlusion, this is one of the big gaps identified in the review. Hybrid frameworks score the best here because, when the camera loses sight of the object, the physics solver continues to simulate the probable trajectory.

While Geometric methods can degrade the object geometry or allow unrealistic self-intersections, Physics-based and Hybrid methods (especially those using PINNs—Physics-Informed Neural Networks) guarantee that the object retains its volume and material properties.

### 5.1. Physical Principles of Flexible Bodies

**Figure 6:**
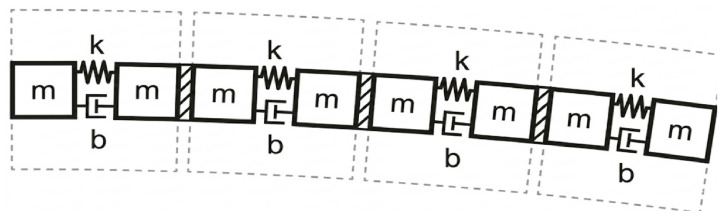
Lumped-parameter model of a flexible beam. The body is divided into mass elements (m) connected by spring (k) and damper (b) elements, forming a simple mass-spring-damper system. Such models illustrate how flexibility can be introduced in multibody simulations, allowing small elastic deformations to be captured (adaptation of Figure 2 from [4]).

Flexible bodies fundamentally differ from rigid bodies in their ability to undergo deformation while maintaining overall structural integrity. In engineering and simulation contexts, a linear flexible body operates in a three-dimensional space where it can undergo large overall motions like rigid bodies (including attachment to other bodies through joints and contact with other objects), but it can also experience deformation under applied forces. This deformation is often represented mathematically through a set of spatial mode shapes and corresponding time-dependent modal coordinates that describe how the object’s geometry changes over time. The physical behavior of these bodies is governed by material properties such as elasticity, mass distribution, and damping characteristics, which collectively determine how the body responds to external forces and constraints (Figure 6). Unlike rigid approximations that assume perfect structural integrity, flexible body models acknowledge that real materials exhibit compliance that can significantly affect system dynamics [4].

The governing equation of motion for a discretized flexible body is expressed in the standard second-order form:

(1) $$M\;ü+C\;\dot{u}\;+K(u)=f\text{\_}\mathrm{ext}(t),$$

where M ∈ ℝ^n×n^ is the consistent mass matrix, C ∈ ℝ^n×n^ is the damping matrix (typically constructed as a Rayleigh combination αM + βK), K(u) ∈ ℝ^n×n^ is the tangent stiffness matrix (nonlinear for large deformations), u, $\dot{u}$, ü ∈ ℝ^n^ are the nodal displacement, velocity, and acceleration vectors, respectively, and f_ext(t) ∈ ℝ^n^ is the vector of external forces at time t.

For linear elastic materials under small-deformation assumptions, K is constant and the system reduces to a linear ODE; for hyperelastic or elastoplastic materials, K(u) must be recomputed at each Newton iteration, contributing the dominant O(n^2^)-O(n^3^) computational cost that constrains real-time feasibility.

Deformation models can be classified by their mathematical formulation and computational approach. Linear deformation models assume that displacements remain small and that the relationship between forces and displacements is linear, making them computationally efficient but limited in representing large deformations. These models are typically used in real-time applications where performance considerations outweigh the need for perfect physical accuracy. Nonlinear deformation models, on the other hand, account for geometric and material nonlinearities, providing more accurate representations of complex behaviors like buckling and large deformations, but at a significantly higher computational cost. Hybrid approaches exist that attempt to balance accuracy and efficiency by combining techniques—for example, treating objects as rigid during free motion, but applying deformation computations during collision events [34]. Additionally, data-driven approaches have emerged that learn deformation behaviors from observations rather than deriving them purely from first principles, potentially offering improved performance for specific classes of objects [35].

### 5.2. Deformation Algorithms and Computational Strategies

Mass-spring systems offer a simpler alternative, modeling deformable objects as networks of point masses connected by springs. Each spring exerts force according to Hooke’s law, leading to intuitive dynamics that approximate soft-body behavior. Mass-spring models are computationally lightweight, though they require careful tuning to ensure stability and may only coarsely approximate real material properties. Techniques such as voxel-based mass distributions and regular grid structures have been used to ensure uniform force distribution and avoid instability in these models [28]. Position-Based Dynamics (PBD) is another popular approach for real-time simulation, especially in computer graphics and game physics. PBD works directly with particle positions, iteratively enforcing constraints (like distances or volumes) to produce physically plausible outcomes. Its stability and speed make it well-suited for interactive applications, even if strict physical accuracy is sometimes traded off. PBD has been widely adopted for simulating cloth, soft bodies, and fluids in real time, given its robustness to large time steps and ease of implementation. Another important concept is the As-Rigid-As-Possible (ARAP) deformation framework, originally developed in geometry processing for shape editing [4]. ARAP enforces that local transformations of a deforming shape remain as close to rigid as possible. This technique has proven particularly effective for non-rigid registration tasks, where a template mesh must deform to fit new data while preserving its intrinsic geometry.

The As-Rigid-As-Possible (ARAP) energy functional is defined as:

E_ARAP(u) = Σ_i_ w_i_ Σ_j_ ∈ N(i) ‖(p_j_ − p_i_) − R_i_ (p′_j_ − p′_i_)‖^2^ (2)

where p_i_, p_j_ denote vertex positions in the reference configuration, p′_i_, p′_j_ denote their deformed counterparts, R_i_ ∈ SO(3) is the local rotation matrix for vertex i, and w_ij_ are cotangent weights for encoding local mesh geometry. Minimization alternates between a local step—computing the optimal R_i_ via singular value decomposition (SVD) for fixed positions—and a global step—solving a sparse linear system for updated positions given fixed rotations. The global step requires factorizing a fixed-sparsity Laplacian matrix, enabling efficient GPU-based solves at interactive rates.

Under Model Order Reduction (MOR), the full displacement vector u ∈ ℝ^n^ is approximated as:

u ≈ Φ q(3)

where Φ = [φ_1_ | φ_2_ | … | φ_k_] ∈ ℝ^n×k^ is the reduction basis matrix whose columns are the k dominant vibration mode shapes (k ≪ n), and q ∈ ℝ^k^ is the vector of modal coordinates. Substituting into the equation of motion and projecting onto the reduced subspace via Φ^T^ yields:

(4) $$\tilde{M}\ddot{q}+\;\tilde{C}\dot{q}+\;\tilde{K}q\;=\;\tilde{f}\text{\_}\mathrm{ext}(t)$$

where $\tilde{M}$ = Φ^T^MΦ, $\tilde{C}$ = Φ^T^CΦ, $\tilde{K}$ = Φ^T^KΦ ∈ ℝ^k×k^ are the reduced mass, damping, and stiffness matrices, respectively. Since k is typically 20–50, this reduces the per-timestep solve from O(n^3^) to O(k^3^), enabling haptic-rate simulation (≥1000 Hz) for objects that would otherwise require several seconds per frame at full resolution.

In systems where efficiency is crucial, additional strategies are used to reduce computational complexity while maintaining essential dynamic properties. One such strategy is Component Mode Synthesis (CMS), which projects deformation onto a reduced basis of vibration modes. By simulating only the dominant modes, CMS can speed up computation with minimal loss of fidelity for small deformations.

**Figure 7:**
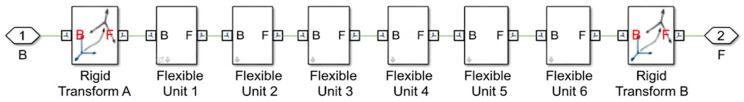
Simscape Multibody model of a flexible structure composed of four “flexible units” connected by rigid transforms. Each flexible unit (encapsulated in a subsystem) contains two mass elements and a joint with an internal spring-damper, approximating a segment of the body (adaptation of Figure 11 from [4]).

Achieving true real-time performance for deformation and reconstruction often requires GPU acceleration at multiple stages of the pipeline—from low-level linear algebra solvers to high-level algorithms for alignment and physics (Figure 7). Modern implementations leverage parallel processing to handle tasks like non-rigid iterative closest point (ICP) alignment and large system solves that would be prohibitive on a CPU [4].

Another common approach is to introduce an initial template acquisition phase: a smooth 3D model of the object is captured under known (often near-rigid) conditions before full dynamic tracking begins. This prior model provides a good starting point and regularization for subsequent non-rigid reconstruction, reducing drift and computational load during live tracking. Finally, hybrid approaches that integrate various modeling techniques can optimize performance by context—for example, computing expensive deformation effects only during collisions or contacts, while treating the object as rigid when in free motion [34]. Exploiting temporal coherence between frames is also key: by using the previous frame’s solution as a warm start for the next, algorithms can greatly reduce the per-frame computation needed.

### 5.3. Physics-Based Methods

Physics-based methods for real-time deformation are grounded in classical mechanics—they simulate how materials respond to forces by solving equations derived from physical principles. These models are valued for providing physically accurate and interpretable results.

Finite Element Analysis (FEA) is a more powerful and general framework for simulating deformable solids. In FEA, the object is discretized into small elements, and the deformation of each element is described by shape functions. The system assembles a large set of equations (typically derived from minimizing an energy function that encodes elastic potential energy) which are solved to find the deformation that balances internal forces and external forces. FEA can accurately model both linear and non-linear material behavior and phenomena like plasticity or fracture. Traditionally, FEA was considered too slow for real-time because solving the large system of equations (especially for 3D meshes with thousands of elements) each time step is computationally expensive. However, there has been progress in model order reduction - reducing the number of active degrees of freedom by precomputing a basis of probable deformations. Techniques like modal analysis yield a small set of mode shapes such that any likely deformation can be approximated by a linear combination of these modes. By simulating only these modes, one can achieve interactive rates. Another approach to speed up FEA is using iterative solvers on the GPU, which can solve the linear systems an order of magnitude faster than CPU for moderately sized problems [20]. As a result, real-time FEM is now feasible for certain applications, especially if approximations are acceptable (e.g., focusing on low-frequency deformations and ignoring very fine details).

Smoothed Particle Hydrodynamics (SPH) is a mesh-free, particle-based method ideal for simulating large or topologically changing deformations in soft tissues and fluids, offering high-fidelity local updates in near real-time through GPU parallelization and localized computational focus [22].

**Figure 8:**
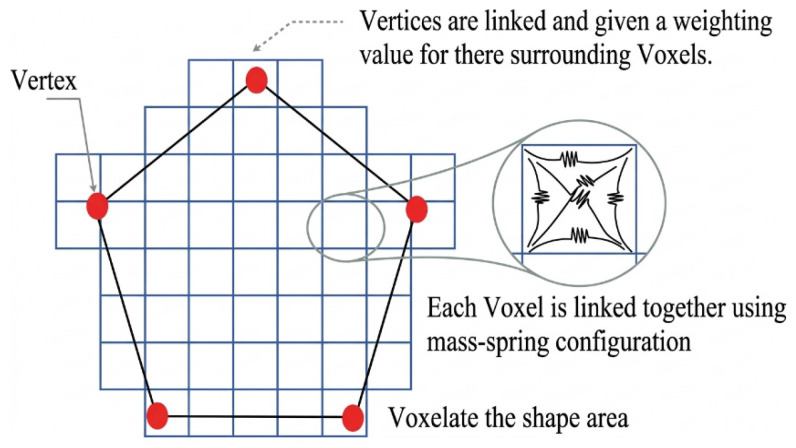
Mass-spring system representation for real-time soft-body deformation, illustrating force distribution across particles using Voxelation Method (adapted Figure 4 from [28]).

Another approach worth mentioning is Position-Based Dynamics (PBD), which, while not strictly derived from physical equations, is often used as a stable physics-inspired solver for real-time applications. In summary (Table 4), physics-based methods like mass-spring, FEM, SPH, and PBD offer a spectrum from simplicity to accuracy (Figure 8). Mass-spring and PBD are simple and fast but require parameter tuning and may deviate from real physics. FEM is accurate but computationally intensive, though reducible with clever optimizations. SPH is flexible and handles extreme deformations but can be slow if not constrained to local regions of interest.

For real-time usage, often a combination of these techniques is used: for instance, a coarse FEM or mass-spring model for global deformation shape, with PBD enforcing positional constraints for stability, and perhaps some SPH particles for special effects like flesh wobbling. Indeed, hybrid physics models are common in practice to leverage the best of each method.

In Position-Based Dynamics (PBD), simulation proceeds by enforcing a set of geometric constraints C_j_(x_1_, …, x_nj_) = 0 directly on particle positions. Given a constraint function C_j_ and current positions x, the position correction Δx_i_ for particle i is computed as:

Δx_i_ = −(w_i_/Σ_j_ w_j_ |∇_xj_ C_j_|^2^)⋯C_j_(x)⋯∇_xi_ C_j_ (5)

where w_i_ = 1/m_i_ is the inverse mass of particle i. This projection step is unconditionally stable and requires no matrix factorization, enabling O(n) per-iteration complexity. The trade-off is that constraint stiffness parameters do not map to physical material constants (Young’s modulus, Poisson’s ratio), limiting quantitative accuracy for material-critical applications such as surgical simulation.

In FEM, the deformation gradient F = ∂x/∂X maps material coordinates X to deformed coordinates x. The Green–Lagrange strain tensor is defined as:

E = ½(F^T^F − I) (6)

For a neo-Hookean hyperelastic material, the strain energy density is:

Ψ(F) = (μ/2)(‖F‖^2^_F − 3) − μ ln(J) + (λ/2)(ln J)^2^ (7)

where J = det(F), μ and λ are the Lamé parameters related to Young’s modulus E and Poisson’s ratio ν by μ = E/[2(1 + ν)] and λ = Eν/[(1 + ν)(1 − 2ν)]. The first Piola–Kirchhoff stress tensor P = ∂Ψ/∂F drives the internal force assembly. For co-rotational FEM, F is factored as F = R S (polar decomposition), and only the symmetric stretch S enters the linear elastic energy, allowing large rigid-body rotations at linear computational cost.

### 5.4. Data-Driven Simulations

Data-driven methods use machine learning, especially modern deep learning, to predict or emulate deformation behavior without the need to solve physics equations directly at runtime. These approaches have seen increasing adoption due to their potential to produce fast (often constant time) predictions after an offline training phase, effectively shifting computational load to a precomputation.

One category of data-driven methods uses supervised learning to train models on pairs of inputs and outputs from simulations or real experiments. For example, a neural network can be trained to take as input the current state of an object (positions of points, or a depth image, etc.) and output the deformed positions after some force is applied. In a manufacturing context, researchers have trained models to predict part deformation during machining processes by analyzing large amounts of data from physics simulations and sensor logs. These models (which could be feedforward neural networks, convolutional neural networks (CNNs) for grid data, or recurrent networks for time series) encode force–deformation relationships for specific object categories and materials.

For a deformable object discretized with approximately 10^3^–10^4^ tetrahedral elements, a full-order implicit FEM solve typically requires 0.5–5 s per frame on a single CPU core, or 50–500 ms on a modern GPU with optimized sparse solvers [20]. A trained neural surrogate operating on the same mesh, such as NNWarp [36] or a GNN-based simulator [37], reduces this to 5–50 ms per frame on equivalent GPU hardware—a speedup of one to two orders of magnitude. For compact latent-space models operating on low-dimensional representations [3], inference times below 1 ms per frame have been reported. These figures are hardware- and problem-specific; for very coarse meshes (<500 elements), FEM and neural methods may converge to comparable inference times, while for high-resolution meshes (>10^5^ elements), neural surrogates maintain their advantage but at the cost of training data requirements that scale with mesh complexity.

It should be noted that this sub-millisecond latency applies specifically to compact, task-specific networks operating on low-dimensional latent representations [3]; more general mesh-based learned simulators, such as MeshGraphNets [37] and GNS [38], typically achieve 20–50 FPS depending on mesh resolution and hardware configuration.

Neural networks are particularly effective at capturing complex non-linear relationships between inputs and outputs that are difficult to model explicitly. They can encode deformation behaviors including elasticity, damping, and even some plasticity implicitly, if those behaviors are reflected in the training data. A deep network can be trained on finite element simulation data for a deformable object under various load conditions. Once trained, it infers the object’s deformation for a new load in a single forward pass. Inference takes milliseconds; equivalent FEM computation requires seconds to minutes. One example in graphics is the concept of subspace neural physics, where a neural network learns to predict the low-dimensional latent variables (mode coefficients) of a reduced physical model, achieving fast and stable updates. Another recent work by Lee et al. introduced a real-time neural cloth deformation model that compresses cloth deformation into a low-dimensional latent space and then uses a small neural network to predict the latent dynamics each frame. This approach was able to produce realistic cloth movement (with folds and wrinkles) at interactive rates, balancing between data-driven speed and physical plausibility [3].

Generative models like Generative Adversarial Networks (GANs) and variational autoencoders (VAEs) have also been applied to enhance realism. For example, once a coarse deformation is obtained (from a physics engine or a simple model), a GAN trained on high-resolution simulations can add plausible high-frequency details such as wrinkles in clothing or small creases in soft tissue. These models essentially learn the space of realistic deformations and can sample from it or interpolate within it to enrich simulation output. There is work on learning deformation fields for video generation (e.g., making a static image move in a realistic way by learning from video data). These generative approaches ensure that the output not only matches the input constraints but also looks realistic overall, having learned implicit physical constraints from data [39].

In Graph Network-based Simulators (GNS) [38], the physical system is represented as a graph G = (V, E) where nodes v_i_ ∈ V encode particle states s_i_ = [x_i_, ẋ_i_, type] and edges e_ij_ ∈ E encode pairwise interactions. Dynamics are learned via iterated message passing:

m_ij_^(l)^ = f_e_(h_i_^(l)^, h_j_^(l)^, r_ij_) (8)

h_i_^(l+1)^ = f_v_(h_i_^(l)^, Σ_j_ m_ij_^(l)^) (9)

where f_e_ and f_v_ are learned edge and node update functions (MLPs), r_ij_ = x_j_ − x_i_ is the relative displacement vector, and l indexes the message-passing iteration. After L iterations, accelerations are decoded as ẍ_i_ = fout(h_i_^(L)^), and positions are updated via a learned Euler integrator. The computational complexity is O(L · |E|), which scales linearly with the number of edges rather than cubically as in FEM, enabling simulation of systems with 10^5^–10^6^ particles at 20–50 FPS [37,38].

In real-time applications, data-driven methods are often used in conjunction with physics-based ones. For example, a neural network might quickly guess the general shape of a deformation, which is then refined by a single iteration of a physics-based solver to enforce any critical constraints. This can drastically cut down the number of solver iterations required, essentially giving the solver a good warm start. Hardware acceleration (using GPUs for the neural network inference) further boosts performance, making it feasible to run complex models each frame.

Data-driven deformation methods face three key limitations:

Generalization: These models interpolate rather than extrapolate—accuracy drops sharply for geometries, materials, or conditions outside the training distribution. A cloth-trained network fails under tool contact or with different stiffness without retraining. This is critical in robotics and surgery, where conditions vary unpredictably. SoftGym benchmarks confirm that learned visual representations underperform ground-truth state inputs on novel conditions [24].

Interpretability: Feedforward nets, CNNs, and GNNs are physically opaque: they produce no accessible representation of stress, strain, or material parameters. Consequently, physically invalid outputs (e.g., self-intersections, volume violations) go undetected internally, and the models cannot be audited—ruling them out for safety-critical applications like surgical guidance or structural assessment [40].

Training costs: Fast inference comes at a heavy upfront price: generating diverse training data requires thousands of expensive simulations, and GNN-based models like MeshGraphNets demand hundreds of GPU-hours [37]. This cost is only justified if the model serves many repeated queries on similar objects—an assumption that breaks down in one-off or highly variable scenarios.

In summary, data-driven deformation methods offer a fast and increasingly accurate alternative to classical simulation, especially tailored to specific objects or scenarios. They have good results in scenarios where lots of prior data is available or where we repeatedly simulate similar objects (so the cost of training is amortized). Their main limitations lie in generalization and the need for training data (which might itself come from many physics simulations or experiments). As computational power grows and machine learning techniques become more sophisticated, data-driven methods are expected to handle more of the deformation workload, potentially learning to simulate materials that are very hard to model from first principles (like biological tissue) by learning directly from observations.

### 5.5. Differentiable and Learned Physical Simulations

Traditional physics simulation is a cornerstone of scientific research, yet it often requires significant domain expertise, meticulous implementation, and massive computational resources [30]. The integration of deep learning with physical principles has introduced new paradigms to address these limitations. Modern approaches typically fall into hybrid strategies that involve learning constitutive models, discretizing governing equations, or optimizing numerical time integration [30]. These advances are particularly crucial for the robotic manipulation of deformable objects (DOM), a field challenged by the infinite-dimensional state space and complex dynamics of materials like cloth, fluids, and soft tissues [30].

Differentiable programming has emerged as a key technology for integrating physical simulators into machine learning workflows [30]. This approach preserves arithmetic intensity and parallelism, often running orders of magnitude faster than traditional frameworks like TensorFlow [30]. Built on similar principles, ChainQueen is a differentiable simulator specifically designed for soft robotics using the Moving Least Squares Material Point Method (MLS-MPM). It allows for real-time inference and gradient-based co-design of robot geometry, materials, and control, significantly outperforming previous non-differentiable soft object simulators [23].

In differentiable simulation, the system state at time t + 1 is computed by a physics step f_θ parameterized by material constants θ (e.g., Young’s modulus, friction):

x_t+1_ = f_θ(x_t_, u_t_). (10)

A scalar loss L(x_1_, …, x_T)—typically a visual reconstruction error or task reward—is backpropagated through the entire trajectory via the chain rule:

∂L/∂θ = Σ_t_ (∂L/∂x_t_)⋯(∂x_t_/∂θ) (11)

where ∂x_t_/∂θ is computed by differentiating through each physics step. This gradient enables direct optimization of physical parameters from raw observational data (video, depth, tactile) without requiring manually specified material constants. The key computational challenge is that ∂f_θ/∂θ involves the inverse of the tangent stiffness matrix K(u), whose computation is the dominant cost in implicit integrators. DiffTaichi addresses this via source-code transformation to generate sparse Jacobians analytically [25], while gradSim employs smooth contact models to ensure differentiability through collision events [26].

Languages like DiffTaichi use source code transformations to generate gradients of simulation steps, enabling the optimization of neural network controllers within just tens of iterations.

Parallel to differentiable programming, researchers have developed learned simulators that utilize Graph Neural Networks (GNNs) to approximate complex physics. The Graph Network-based Simulator (GNS) framework represents physical states as interacting particles and computes dynamics via learned message-passing (Table 5). GNS is remarkably versatile, capable of simulating fluids, rigid solids, and deformable materials within a single architecture, while generalizing to trajectories much longer than those seen during training [29]. Expanding this to mesh-based representations, MeshGraphNets allows for learning resolution-independent dynamics on adaptive meshes. This framework is highly efficient for high-dimensional tasks such as aerodynamics and structural mechanics, running up to two orders of magnitude faster than the ground-truth solvers on which it was trained [37].

The technical properties of these differentiable frameworks—gradient propagation through physics engines, parameter identifiability, and computational efficiency—carry direct implications for the research gaps identified in this review. These implications are examined in depth in Section 9.4.

Data-driven models, including Jacobian-matrix-based and GNN-based models, offer stronger representation power but require substantial datasets.

Differentiable simulators carry four notable limitations:

Memory and computing: Backpropagating through T timesteps requires O(T·n) memory, making full trajectories prohibitive for large meshes. DiffTaichi [30] reduces this via sparse Jacobians but struggles beyond ~10^4^ elements; gradSim [26] and DPSI [41] similarly cannot reach surgical-resolution meshes without checkpointing that increases runtime.

Ill-posedness and local minima: Recovering material parameters from observed trajectories is inherently ill-posed—multiple parameter sets can match noisy or sparse observations equally well. Gradient-based optimization is initialization-sensitive and prone to local minima that look visually correct but are physically wrong [26,41], a problem amplified by heterogeneous materials with high-dimensional parameter spaces.

Contact modeling bias: Differentiability through collisions requires smooth approximations of contact and friction, introducing systematic error: the simulator represents a regularized surrogate, not the true system. For dense contact, sharp material interfaces, or topological changes (tearing, cutting), this approximation degrades gradient accuracy [26,30].

Limited generalization: Parameters identified from a short interaction sequence may not transfer to different loading conditions or contact geometries. DPSI [41] shows strong held-out performance on the same object but degraded accuracy under substantially different forces or contacts, reflecting the fundamental identifiability limits of inverse problems with sparse observations.

## 6. Applications of Flexible Body Modeling

### 6.1. Research Motivation and Applications

The accurate modeling of flexible bodies addresses fundamental limitations in traditional rigid-body assumptions that have constrained technological advancement across multiple fields. In robotics, the prevailing assumption of rigidity has historically limited the ability of robots to interact with deformable materials that characterize many real-world environments [1]. While rigid-body models are adequate for highly controlled settings, they break down in dynamic scenarios where objects exhibit compliance. Deformable materials present unique challenges due to their infinite-dimensional and non-linear nature, which drives research into new methods for representation and simulation.

By enabling robots to understand and predict deformations, flexible body modeling can improve robotic manipulation of soft objects, leading to advances in areas like grasping delicate items or human–robot interaction with safe, compliant actuators [4].

Beyond robotics, flexible body modeling meets critical needs in other domains. In aerospace engineering, for instance, accounting for wing flexibility is essential because deformation can significantly impact flight dynamics (e.g., aeroelastic phenomena like wing flutter). In civil engineering, structural flexibility must be accurately predicted to ensure safety and performance under loads such as wind or seismic activity. Biomedical engineering also benefits from deformable models, where representations of soft tissue can improve surgical simulators and prosthetic design by reflecting realistic tissue behavior. The integration of real-time deformation capabilities into interactive systems enables new perspectives for human–computer interactions, training simulators, and design tools. For example, surgical training simulators with real-time deformable tissue models provide realistic haptic feedback, allowing practitioners to develop skills in a safe virtual environment. In virtual design and prototyping, engineers can manipulate digital models of flexible materials (like bending a virtual cable or sheet metal) to immediately see the results of their actions, improving the design workflow.

### 6.2. Virtual and Augmented Reality Implementations

**Figure 9:**
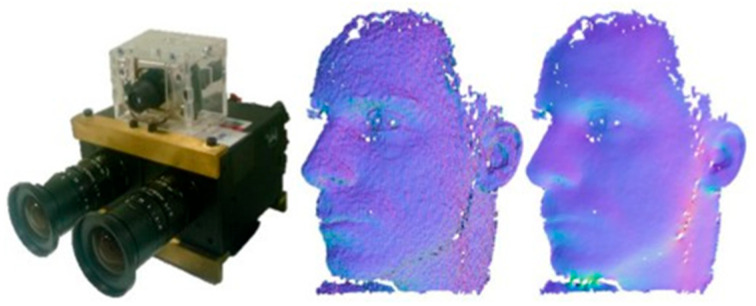
Real-time non-rigid reconstruction in virtual environments, enabling realistic interactions with deformable objects. Left to right: our active stereo sensor, the PatchMatch result, and the result after variational refinement (taken from [35]).

In virtual reality (VR), convincing immersion depends on the realism of interactions with the virtual world. The inclusion of physics-based interactions with deformable virtual objects greatly enhances this realism. Users can squeeze, bend, or stretch virtual objects and see them respond just as physical objects would, which improves the sense of presence (Figure 9).

Such accurate tissue deformation is critical for developing surgical skills in a safe environment. Another advantage is the reconstruction of avatars and virtual environments. Recent research has demonstrated capturing a user’s body, including fine clothing wrinkles and body deformations, and injecting that live model into VR [34]. In augmented reality contexts, understanding the deformation properties of real-world objects allows virtual elements to interact more convincingly with the physical environment, such as virtual water flowing realistically over deforming surfaces or virtual characters that respond appropriately when interacting with flexible physical objects. These applications benefit particularly from recent advances in marker-less reconstruction using consumer-grade hardware, making sophisticated deformation modeling accessible without specialized equipment.

Marker-less reconstruction using consumer-grade hardware (like a single depth camera or even just RGB cameras with the help of machine learning) has brought some of these advanced capabilities within reach. We now see demonstrations of a person wearing a VR headset and holding a real object (like a foam ball); the system tracks and reconstructs the foam ball as it is squished in the user’s hand and mirrors that deformation on the virtual ball seen in VR. This blending of real and virtual via deformable object tracking constitutes a technically significant capability for AR/VR applications.

### 6.3. Robotics and Automated Manufacturing

In robotics, especially in unstructured environments, robots frequently encounter deformable objects. Traditional robotics algorithms often assumed rigid objects for simplicity, but this limits the robot’s ability to deal with the real world. Deformation modeling in robotics helps the robot predict how an object will change shape when grasped or manipulated, leading to better planning and control. For instance, when a robot wants to pick up a deformable object like a bag of fluid or a piece of fabric, it can simulate various grasping strategies to see which causes minimal undesirable deformation (spillage or creasing).

Grasp planning for deformable objects is an active area of research; planners incorporate deformable object simulators to evaluate candidate grasps. Some approaches break the problem first reconstructing the object’s shape in 3D, then using a physics model to simulate the outcome of a grasp. Without understanding deformation, a robot might grasp a T-shirt in a way that it slips out or folds in a troublesome manner. With deformation awareness, the robot can choose a grasp that leads to, say, the T-shirt hanging open for easy folding.

Soft robotics is another subfield intimately tied to deformation. Soft robotic arms or grippers are themselves made of flexible materials. Real-time reconstruction of a soft robot’s shape (via embedded sensors or external cameras) can serve as feedback to its control system. In contrast, it is essential to simulate the deformation of a soft robot during actuation and contact to control it.

**Figure 10:**
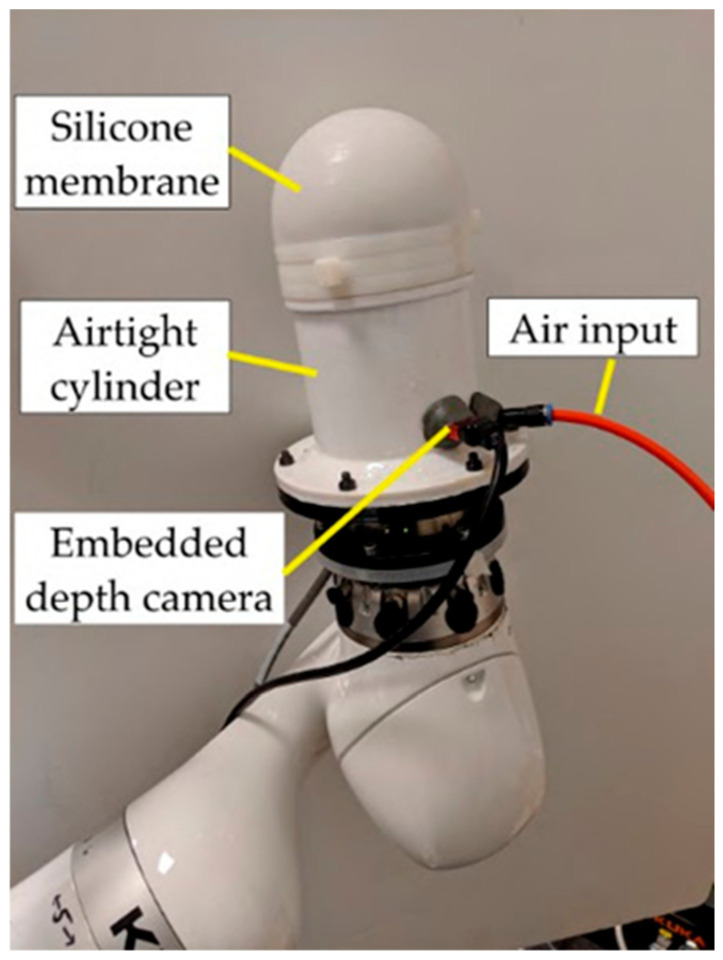
Soft tactile sensor used for robotic manipulation of deformable materials in automated manufacturing contexts (taken Figure 2.2 from [1]).

The synergy between sensing and deformation simulation allows soft robots to adapt to complex tasks, like gently conforming to a human’s body to aid or safely interacting in cluttered environments by deforming around obstacles. In automated manufacturing processes, robots are increasingly required to handle non-rigid parts. Many modern robots include soft tactile sensing pads that measure contact forces and can even estimate the shape of the contact surface by how they deform (Figure 10).

### 6.4. Medical Imaging and Simulation

Medical applications of real-time deformation modeling are wide-ranging, spanning from diagnostic imaging to surgical planning and training. In many medical scenarios, soft tissues deform significantly, and understanding or compensating for these deformations can be the difference between success and failure.

In medical imaging, one challenge is that human tissues move and deform between or during imaging sessions. For instance, comparing a pre-operative scan of a liver to an intra-operative situation requires accounting for breathing motion or surgical manipulation that deform the liver. Non-rigid image registration techniques align images taken at different times or with different modalities by allowing deformable transformations. Real-time 3D reconstruction techniques can track organ surfaces (using ultrasound or stereo cameras in the operating room) and register them to pre-op 3D models (from CT/MRI), updating the model to the current shape. This is critical for intraoperative guidance. A tumor marked pre-operatively must be localized in the current anatomy after organ shift and deformation. The pre-operative image is warped to match the patient’s current state.

**Figure 11:**
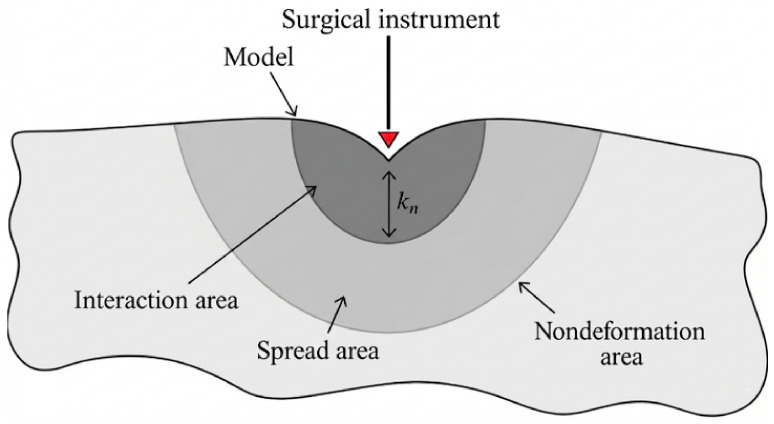
Smoothed Particle Hydrodynamics (SPH) simulation of soft tissue deformation under an applied force, used for a surgical training scenario. Particles represent tissue material points; their movement and interaction simulate how soft tissue yields to a tool in real time (adaptation of Figure 4 from [22]).

Surgical simulation is a major area benefiting from deformation modeling. Virtual reality-based surgical trainers allow surgeons to practice procedures on a computer-generated patient. For realism, these systems simulate deformable anatomy—skin, muscle, organs—with real-time physics so that when a surgeon-in-training uses virtual instruments on them (with a haptic interface for force feedback), it feels and looks authentic. For example, a needle insertion simulation needs to model how tissue stretches and yields under the needle, and how it closes around it. These simulations often use FEM or mass-spring models optimized to run at haptic rates. They are particularly valuable for minimally invasive surgery training (like laparoscopy), where direct vision is limited and surgeons must rely on understanding how tissues deform under their tools indirectly (Figure 11).

The application domains surveyed above—virtual and augmented reality, robotics, and medical simulation—share a common requirement for reliable, quantifiable performance.

The following section examines how methods across these domains are evaluated, reviewing the datasets, metrics, and benchmark frameworks currently used to assess reconstruction accuracy and deformation realism.

## 7. Evaluation & Benchmarking

### 7.1. Datasets and Benchmarks for Flexible-Body Deformation

Robust evaluation of 3D reconstruction and real-time deformation methods for flexible bodies critically depends on the availability of realistic, well-documented datasets and benchmarks. While recent years have seen a marked increase in datasets targeting specific aspects of deformable-object perception, such as non-rigid structure—from-motion, implicit dynamics, or visuo-tactile interaction—the overall ecosystem remains fragmented. Most works still rely on self-recorded sequences or narrowly scoped synthetic environments, which hampers systematic comparison across methods and application domains. This chapter reviews the main families of datasets used in the literature and highlights the aspects of deformable behavior they capture, as well as the important gaps that remain.

### 7.2. Non-Rigid Structure-from-Motion and Shape-from-Template

Table below (Table 6) provides a structured comparative overview of the principal datasets currently used for evaluating flexible-body reconstruction and deformation methods, organized by evaluation target, data modality, availability of ground-truth physical parameters, and support for contact-rich or multi-object scenarios.

However, even this benchmark concentrates primarily on sparse or semi-dense point trajectories and surface reconstructions rather than full volumetric dynamics or contact-rich interactions. As a result, it is well suited to evaluating geometric tracking performance but less informative about physical realism, long-horizon dynamics, or the behavior of methods in heavily occluded, multi-object scenes.

### 7.3. Simulation-Based and Interaction Benchmarks

To study action–response relationships and long-horizon behavior under controlled physical parameters, several works introduce simulation-based datasets. ACID defines an action-conditional implicit dynamics model for volumetric plush toys in NVIDIA Omniverse and is trained on over 17,000 action trajectories collected from six families of stuffed objects (78 variants) [43]. The associated PlushSim environment and dataset have become the standard benchmark for implicit visual dynamics of semi-stiff, elastically deformable objects [43].

Complementary approaches, such as NeuSpring, focus on reconstructing and simulating deformable objects from RGB-D video and evaluate cloth, rope, and packaging scenarios using a spring-mass formulation with learned physical parameters. These simulation-based datasets are particularly useful for benchmarking model-based and learned dynamics, analyzing stability and convergence of real-time integrators, and probing the identifiability of material parameters. Their main limitations lie in the visual domain gap to real data and, in some cases, restricted diversity of geometries and interaction patterns [31].

A complementary family of benchmarks extends this line of work toward contact-rich scenarios, where accurate modeling of hand–object interaction, occlusion, and multi-object configurations become equally important.

DexYCB provides a large-scale dataset of human hands manipulating rigid and articulated objects but does not include deformable targets. To address this gap, ViTaM-D introduces the HOT dataset, which contains 600 sequences of dexterous interaction with 30 deformable objects across five categories, captured in the ZeMa physics-based simulator using finite element models and multi-view RGB-D streams combined with distributed tactile arrays. HOT enables systematic assessment of reconstructed hand–object geometry, contact patches, and penetration depth under controlled deformations [32].

Real-world visuo-tactile corpora further extend this line of work. For example, datasets recorded with a stretchable, 1152-channel tactile glove and multi-viewpoint clouds capture contact-rich manipulation of both rigid and elastic objects, such as sponges, slime, and bags. These resources support joint evaluation of deformation reconstruction and contact localization under severe occlusions. Nevertheless, limitations remain in terms of the diversity of human motion, object categories, and environmental conditions represented, as well as the complexity of sensor calibration and synchronization [44].

### 7.4. Digital-Twin and Application-Specific Case Studies

Beyond generic benchmarks, several works introduce application-specific datasets as part of integrated “modeling–rendering–simulation” pipelines, often under the digital-twin paradigm. In minimally invasive surgery, for example, EndoNeRF-style reconstructions of deforming soft tissue have been combined with finite element or material point method (MPM) simulation to build soft-tissue digital twins that support training and planning. Other studies combine 3D Gaussian Splatting with particle-based elastodynamics to realize high-fidelity, real-time digital replicas of deformable environments [2,45].

These case studies typically offer rich, multi-modal information and realistic, domain-specific scenarios, but they are tailored to narrow use cases and are rarely adopted as general-purpose benchmarks. Evaluation protocols, metrics, and task definitions vary significantly across works, which limits their role as standardized reference points for the broader community [45].

### 7.5. Cross-Cutting Challenges

A structured analysis of the datasets reveals four cross-cutting gaps that together explain why a fair and reproducible comparison between the method families in Figure 5 remains impossible with currently available resources.

Gap 1—Coverage of evaluation dimensions: No single dataset simultaneously provides dense geometry ground truth, long-horizon dynamics, verified material parameters, and contact fidelity data. The NRSfM Animatronics benchmark [42] offers the most systematic geometric ground truth but contains no physical parameters and no contact scenarios. HOT [32] provides contact patches and FEM-derived ground truth but is limited to hand-sized objects and specific deformation categories. SoftGym [24] enables standardized RL benchmarking but relies on PBD constraints that do not map to real material constants. This fragmentation means that a method optimized for one evaluation dimension—say, geometric tracking accuracy on the Animatronics benchmark—cannot be straightforwardly compared with a method optimized for contact fidelity on HOT, even if both claim to address flexible-body reconstruction.

Gap 2—Evaluation protocol heterogeneity: Across the 56 included works, at least eight distinct primary metrics are used: Chamfer Distance, endpoint error, PSNR/SSIM, contact-IoU, penetration depth, task success rate, mean surface distance, and F-score. Because different works select different subsets of these metrics—often the subset most favorable to their method—cross-study comparison is severely limited. Furthermore, evaluation hardware, object size ranges, and deformation magnitudes are rarely standardized, so even when the same metric is reported, the numbers are not directly comparable.

Gap 3—Synthetic-to-real domain gap in available datasets: The majority of large-scale datasets with dense ground truth—including ACID [43], SoftGym [24], and the gradSim evaluation sequences [26]—are generated in physics simulators. While this enables controlled evaluation of specific hypotheses, it introduces a systematic domain gap: methods that perform well on synthetic benchmarks may fail on real sensor data due to lighting variation, sensor noise, and the inaccuracy of the simulator’s constitutive model relative to real material behavior. Of the datasets reviewed, only the Animatronics benchmark [36], the stretchable tactile glove corpus [44], and the HOT dataset [32] provide real-world capture with verified ground truth.

Gap 4—Limited accessibility: Of the ten datasets catalogued in Table 6, only four are fully publicly available without restrictions. The remainder are available upon request or in partial form, which limits reproducibility and prevents their adoption as community-wide reference benchmarks. This accessibility barrier is particularly significant for surgical datasets such as EndoNeRF [2], where patient privacy constraints further restrict distribution.

### 7.6. Summary and Open Issues

Taken together, existing datasets provide valuable testbeds for particular aspects of deformable-object reconstruction and dynamics: NRSfM benchmarks emphasize geometric tracking; simulation-based datasets such as ACID and NeuSpring focus on controllable dynamics and material parameters; visuo-tactile corpora and HOT target contact-rich hand–object interaction under occlusion; and digital-twin case studies demonstrate high realism in specific medical or industrial domains.

However, the field still lacks a single “gold-standard” benchmark that jointly covers dense geometry, long-horizon dynamics, contact fidelity, and task performance across a diverse set of flexible bodies. This fragmentation directly impacts several of the challenges discussed later in the survey, particularly the difficulty of comparing speed–accuracy trade-offs and occlusion robustness across methods, and the limited transferability of results between application domains. Addressing these limitations will require coordinated community efforts to design physics-aware, multi-modal benchmarks with standardized evaluation protocols that explicitly target deformable objects in realistic, task-driven scenarios.

In contrast to prior work, which typically treats geometric reconstruction, physical simulation, and data-driven modeling as separate problems, this study provides a unified perspective on deformable object modeling by systematically analyzing their interdependencies.

Specifically, this work bridges the gap between geometry-based reconstruction and physically plausible deformation by examining how reconstruction accuracy, material modeling, and interaction dynamics jointly influence system performance.

Rather than focusing on a single methodological paradigm, we identify the trade-offs between physics-based, data-driven, and hybrid approaches, highlighting their respective strengths and limitations in real-time and dynamic scenarios.

Furthermore, we emphasize the role of multimodal sensing and benchmark fragmentation as key factors that currently hinder fair evaluation and real-world deployment.

By synthesizing insights across these domains, this survey provides a structured framework for understanding the design space of deformable object reconstruction and simulation systems.

A viable physics-aware benchmark must jointly satisfy five requirements: (1) multi-modal ground truth—dense geometry, verified material parameters (Young’s modulus, Poisson’s ratio, density), and contact force distributions; (2) diverse object categories (cloth, soft tissue, cables, elastomers) with intra-category variation; (3) standardized metrics, hardware reference, and normalization for cross-method comparability; (4) both lab and real-world sequences to quantify the sim-to-real gap; (5) public availability under a permissive license.

No existing dataset meets all five jointly, though NRSfM Animatronics [42], HOT [32], and PlushSim [43] offer partial building blocks. Constructing this benchmark—analogous in impact to ImageNet for visual recognition—is the field’s highest-leverage investment, requiring coordinated effort across computer vision, robotics, and computational mechanics.

Finally, we outline open challenges and promising research directions toward the development of unified, scalable, and physically consistent models capable of operating in complex, real-world environments.

Building on the gap analysis above, a minimally viable physics-aware benchmark for this field would need to satisfy five requirements that no existing dataset currently meets jointly. First, it must provide multi-modal ground truth combining dense geometry (structured-light or stereo), verified material parameters (Young’s modulus, Poisson’s ratio, density) obtained through independent mechanical testing, and contact force distributions from calibrated tactile arrays. Second, it must include a diverse set of flexible body categories spanning at least cloth, soft tissue analogues, cables, and foam-like elastomers, with sufficient intra-category variation in geometry and material properties to support generalization assessment. Third, it must define a standardized evaluation protocol specifying the metrics, hardware reference platform, and object size normalization required for cross-method comparability. Fourth, it must include both controlled laboratory sequences and unstructured real-world sequences to enable quantification of the synthetic-to-real gap. Fifth, it must be fully publicly available under a permissive license to enable broad community adoption.

The construction of such a benchmark constitutes a significant but tractable community effort, analogous in scope to the role that ImageNet played in standardizing evaluation for visual recognition. Several existing initiatives—the NRSfM Animatronics benchmark [42], the HOT dataset [32], and the PlushSim environment [43]—provide partial building blocks that could be extended and integrated toward this goal. Coordinating this effort across the computer vision, robotics, and computational mechanics communities represents, in the view of this review, the single highest-leverage investment the field could make toward enabling reliable, reproducible progress.

### 7.7. Evaluation Metrics for Deformation Realism

Most works discussed in this review resort to geometric measures such as Chamfer distance (CD) and endpoint error (EPE) between reconstructed and ground-truth point clouds. While these metrics are indispensable for quantifying reconstruction accuracy, they only partially capture how “realistic” a deformation is in a physical or perceptual sense. Recent papers therefore augment CD/EPE with more specialized metrics, which can be grouped into robust geometric, contact-aware, physics-aware, task-level, and image-space criteria (Table 7).

The NRSfM benchmark of Jensen et al. introduces a robust 3D error metric that down-weights large outliers based on robust statistics, thereby reducing sensitivity to a few badly reconstructed frames or points [42]. This approach complements standard RMS distances and provides a more reliable summary of behavior over long sequences. Related works also evaluate dense non-rigid reconstruction using symmetric surface distances such as average surface distance and Hausdorff distance, particularly in biomedical contexts where surface over- or under-estimation is clinically critical [2].

In applications where contact fidelity is crucial, several authors explicitly measure the plausibility of hand–object or tool–tissue interactions. ViTaM-D evaluates reconstructed hand–object interactions on HOT using two metrics: (i) maximum and average penetration depth between reconstructed hand meshes and deformable objects; and (ii) contact-IoU between predicted and ground-truth contact regions, defined as surface points within 2–3 mm of the opposing mesh [32]. Lower penetration and higher contact-IoU indicate more physically plausible interactions, even when global shape errors are small. Similarly, visuo-tactile methods that jointly estimate object deformation and contact patches use the distance between predicted contact points and the true object surface as an asymmetrical measure of whether the inferred contact is physically feasible [2].

Differentiable system-identification frameworks such as gradSim and DPSI investigate not only visual similarity but also the consistency of recovered physical parameters and stress fields [26,41]. DPSI, for example, compares simulated and real deformations of elastoplastic blobs using both point-cloud distances (CD/EMD) and height-map discrepancies over the support surface, and analyses how well the estimated Young’s modulus, yield stress, and friction coefficients reproduce unseen long-horizon interactions [41]. In generative modeling of physically plausible shapes, works like Phy3DGen and PhysComp3D incorporate stress-based objectives into their loss functions, explicitly encouraging generated objects to exhibit uniform or mechanically admissible stress distributions under prescribed loads [45,46]. These physics-aware criteria move beyond purely geometric alignment and directly assess whether the deformation would be consistent with continuum mechanics.

**Table 7:** Benchmark frameworks for deformable object learning.

Feature	Core Technology	Primary Focus	Data Representation	Optimization Goal	Key Advantage	Speed vs. Ground Truth
DiffTaichi [30]	Differentiable Programming	High-performance gradients	Global Tensors/Arrays	Controller/Parameters	High parallelism (188× vs. TF)	Comparable to CUDA
ChainQueen [23]	MLS-MPM Differentiable	Soft Robotics	Particles and Grid nodes	Design and Control	Real-time soft object co-design	4–9× faster than state-of-art
DOM Survey [47]	Data-Driven/Analytical	Robotic Manipulation	Various (Particles, GNN)	Task Planning	Comprehensive roadmap	N/A
SoftGym [24]	RL Benchmark (FleX)	Deep RL Benchmarking	Particles/Constraints	Reinforcement Learning	Standardized reproducibility	4× faster than real-time
Hybrid DL [29]	Hybrid DL/Physics	Simulation Paradigms	Meshes/Particles	Numerical Efficiency	Systematic classification	Varies by hybrid strategy
MeshGraphNet [37]	Mesh-based GNN	Aerodynamics/Structural	Adaptive Meshes	Forward Dynamics	Resolution independent	10–100× faster
GNS [38]	Particle-based GNN	Multi-material Physics	Unstructured particles	Forward Trajectories	Generalizes to large systems	Comparable to GT

For manipulation and dynamics models, realism is increasingly evaluated through task outcomes and point-wise correspondences. ACID reports, in addition to Chamfer distances, the F-score and volumetric IoU between the achieved and target shapes in goal-conditioned manipulation tasks, as well as the mean-squared error of dynamics predictions and the recall of learned dense correspondences across large non-rigid motions [48]. These metrics quantify whether the learned implicit dynamics are realistic enough to support successful reconfiguration of volumetric deformable objects under complex contact sequences.

NeRF-based reconstruction of deforming soft tissues in surgery (e.g., FastEndoNeRF) and world-model approaches evaluate visual realism using photometric metrics such as peak signal-to-noise ratio (PSNR), structural similarity (SSIM), and perceptual distances (e.g., LPIPS) between rendered and real endoscopic frames [2,46]. These measures are especially important when the primary consumer is a human operator (surgeon, remote collaborator, or VR/AR user), and they complement geometric metrics that may be unavailable in vivo.

Overall, the recent literature confirms a trend from simple point-wise distances towards composite evaluation protocols that integrate geometric accuracy, contact fidelity, physical consistency, task performance, and visual plausibility [32,42,48]. This trend is in line with the research gaps highlighted in this work regarding inadequate physical modeling and the computational burden of physics-based methods: more nuanced metrics are necessary to fairly assess methods that trade off high-fidelity physics against real-time performance.

The evaluation landscape reviewed above makes clear that current datasets and metrics, while improving, still leave significant gaps in how we measure physical plausibility, occlusion robustness, and multi-object generalization.

These measurement gaps mirror deeper methodological limitations that the community has yet to resolve.

The following section examines the four systemic challenges that persist across the literature, along with concrete research directions that could address them.

## 8. Challenges and Future Research Directions

Real-time 3D reconstruction of non-rigid objects is a challenging problem at the intersection of computer vision and graphics. Unlike rigid structure-from-motion, deformable scene reconstruction is an inverse problem that lacks a unique solution due to the complexity and flexibility of the scene: without strong prior assumptions, there are infinitely many 3D shapes that can explain the same 2D images.

Modern approaches have achieved substantial quantitative progress in tracking and modeling deformable surfaces from monocular video or depth streams (Table 8). On the NRSfM Animatronics benchmark [42], state-of-the-art methods report mean 3D reconstruction errors below 5 mm for bending and stretching scenarios, compared to errors exceeding 20 mm for classical low-rank NRSfM methods on the same sequences. NeRF-based reconstruction of deforming soft tissue achieves PSNR values of 28–34 dB on endoscopic video sequences [2], approaching the quality of multi-view reconstruction at a fraction of the hardware cost. Despite this progress, performance degrades significantly under severe occlusion, fast motion, and topology changes—conditions that are common in unstructured real-world deployment but underrepresented in current benchmarks, as discussed in Section 7.5.

Guided by a scoping-review methodology, we identify four systemic shortcomings that persist across literature:

(i) The absence of a unified, physics-aware benchmark for deformable objects.
(ii) Enduring speed–accuracy trade-offs that hinder deployment in complex real-world scenarios.
(iii) Fragile handling of occlusions, multi-object interactions, and physical plausibility; and
(iv) Limited multimodal sensor integration in end-to-end reconstruction–simulation pipelines: These gaps are particularly critical for applications in robotics, where safe and reliable interaction with deformable materials depends on both accurate geometry and trustworthy dynamics, and in medicine and AR/VR, where visual fidelity must be aligned with physically plausible behavior.

### 8.1. Speed vs. Accuracy Trade-Off

Achieving both high reconstruction accuracy and real-time speed remains a central tension in non-rigid 3D reconstruction. Methods that prioritize accuracy often perform expensive optimization or use complex deformation models, which slow down processing. Conversely, systems optimized for real-time performance tend to simplify the model or use fewer iterations, degrading the quality or detail of the reconstructed geometry. In practice, real-time performance comes at the cost of noticeably lower reconstruction quality. This trade-off affects deployment: for instance, in an AR/VR application or robotic system that demands instantaneous feedback, one might have to tolerate drift, lower mesh resolution, or lag in capturing fast deformations. On the other hand, high-accuracy offline methods cannot be used in live scenarios. Thus, balancing speed and accuracy is a persistent gap—current algorithms often must choose one over the other, limiting their usefulness in scenarios that require both precision and interactivity.

It should be noted that the speed–accuracy figures cited throughout this section are drawn from heterogeneous experimental conditions across the 56 included works, as catalogued in Table 6. The absence of a unified benchmark with standardized hardware and evaluation protocols means that these figures cannot be directly compared and should be interpreted as indicative ranges rather than definitive rankings. Establishing such a benchmark, as discussed in Section 7.6, is a prerequisite for the quantitative resolution of this trade-off.

One promising direction is to design multi-resolution and adaptive algorithms that allocate computational effort strategically. By using coarse-to-fine reconstruction pipelines, a system can perform quick, global alignment at a low resolution and then refine local details only where needed. Such hierarchical approaches maintain real-time frame rates by avoiding unnecessary computation on well-predicted regions while still preserving accuracy on fine structural details. For example, a reconstruction algorithm could run a fast initial pass to estimate the bulk deformation and then iteratively refine areas with high error using additional local iterations or higher mesh density. This dynamic allocation of effort helps to reconcile the speed–accuracy conflict, ensuring that simpler parts of the scene do not bottleneck the overall processing.

Another opportunity lies in leveraging data-driven priorities and hardware acceleration to speed up complex computations. Machine learning models (such as deep neural networks) can be trained to predict deformation updates or key point correspondences in a single feed-forward pass, effectively bypassing some of the costly iterative optimization at runtime [40]. These learned components act as intelligent predictors that give the reconstruction a “head start,” which can then be fine-tuned by a lightweight optimization step. Combining learning-based predictions with traditional model-based refinement can substantially reduce processing time with limited loss of accuracy. In parallel, exploiting modern hardware—GPU parallelism, dedicated depth processing units, and even specialized ASICs—can accelerate linear algebra and physics computations that are the core of many reconstruction algorithms. For instance, tailored GPU implementations of bundle adjustment or non-linear solvers have been shown to achieve real-time convergence by fully utilizing the sparsity patterns in the problem [35]. Embracing these optimizations and co-designing algorithms with hardware in mind will allow future systems to narrow the gap between fast and accurate non-rigid 3D reconstruction.

### 8.2. Occlusions, Viewpoint Ambiguity, and Multi-Object Scene Handling

Occlusions and limited viewpoints introduce significant ambiguity into deformable reconstruction.

When a flexible object is observed by only a single camera or a narrow set of views, parts of the object are inevitably self-occluded or outside the field of view.

The system must then deduce the shape and motion of these unseen regions, which is fundamentally difficult.

In monocular non-rigid reconstruction, the problem is ill-posed: many different 3D shapes can produce the same 2D projection, and occlusions exacerbate this by removing direct observations of large portions of the surface.

Single-view systems can accumulate errors over time, especially when parts of the object move in and out of sight, potentially producing ghost artifacts or gross shape errors, or failing to track the object when occlusion is prolonged.

Multi-view setups reduce this problem by providing complementary viewpoints, yet inter-camera occlusions—where a surface part is hidden from all cameras simultaneously—can still cause ambiguous shape estimates.

These limitations are particularly consequential in unstructured environments where a deformable object interacts with other objects or a user, creating frequent occlusions that break many current algorithms.

Occlusion problem solving needs to involve both hardware techniques and algorithms. Light projectors reduce the effect of occlusion through providing additional data sources which can be fused in real-time, although this approach adds some complexity of calibration and synchronization [46]. A specific system-level response to occlusion is OcclusionFusion, which explicitly identifies occlusion boundaries in the depth image, maintains a reconstruction that avoids erroneously carving occluded space, and reintegrates surface data as regions become visible. On the algorithmic side, even single-view systems can incorporate statistical shape models or learn spatiotemporal priors to infer the likely geometry of hidden parts. By learning how deformations propagate across a surface, graph neural networks and recurrent models can predict the motion of currently occluded regions from the motion visible elsewhere. Equipping such systems with explicit uncertainty estimates for occluded regions—allowing a robot to reposition for a better view when uncertainty is high—is a promising direction for closing the gap between occlusion-robust inference and reliable real-world deployment.

On the hardware side, deploying multiple cameras or depth sensors is the most direct approach, as each additional viewpoint increases coverage and reduces blind spots.

A closely related and compounding challenge is the handling of scenes that contain multiple deformable objects or a flexible target interacting with a cluttered background.

Most existing non-rigid reconstruction methods assume a single, isolated deforming object and assign all observed motion to one coherent model.

In more realistic scenarios, this assumption breaks down: multiple flexible objects can move independently, occlude one another, and exchange contact forces, while background clutter introduces spurious depth measurements.

Without explicit multi-object capability, algorithms tend to merge all motion into a single model, drop one object when two surfaces separate after contact, or fail to assign surface data correctly to the responsible body.

A practical illustration is a person holding a deformable prop such as a bag or cloth: current systems often either focus on the person and discard the deformable object, or vice versa, unable to maintain both models simultaneously.

Addressing multi-object scenes requires extending both scene representations and tracking formulations.

One direction is to integrate a dynamic SLAM-style framework that maintains a static background map alongside separate deformable models for each moving object, estimating camera motion, background geometry, and individual object deformations jointly.

Methods such as multi-body NRSfM [11] take a step in this direction by using group dictionary learning to reconstruct multiple interacting bodies from uncalibrated multi-view images, though they remain largely offline.

An equally important requirement is physically consistent collision handling: when two deformable objects make contact, the reconstruction should enforce non-penetration constraints and model force exchange, rather than allowing surfaces to pass through one another.

Embedding collision detection and response directly into the reconstruction optimization is computationally demanding in real time but constitutes a necessary step toward pipelines capable of faithfully representing complex, contact-rich environments—a prerequisite for reliable robotic manipulation and surgical simulation applications.

### 8.3. Inadequate Physical Modeling

A notable gap in current real-time non-rigid reconstruction systems is the lack of true physical modeling of material properties and dynamics. Most existing methods rely on geometric constraints or simple smoothness prior rather than enforcing actual physical laws of the objects. For example, many reconstruction algorithms impose that the surface should deform smoothly and maybe preserve local distances (ARAP), but they do not include properties like stiffness, mass, or internal forces that govern real deformations. As a result, reconstructions may be kinematically plausible (they fit the images) but not physically accurate. A reconstructed cloth might appear to flap correctly in the video, but the inferred motion might imply it is lighter than air or infinitely stretchable—inconsistencies that are hidden unless one tries to apply new forces to the model.

This lack of physical grounding limits certain applications. If the goal is not just to reconstruct but also to simulate or predict behavior under new conditions, a model that has no notion of real forces is insufficient [40]. For instance, if we want to use a non-rigid reconstruction of an organ during surgery to predict how it will move when a tool pushes it, then a purely geometric reconstruction provides no answer—we need a physical model of tissue elasticity. Similarly, in AR applications, if virtual objects are to interact realistically with a reconstructed deformable object (say, a virtual ball bouncing on a real trampoline), the system must know the real physical properties (the trampoline’s tension, damping, etc.) to simulate that interaction believably.

One way to bridge this gap is to integrate physics-based constraints and models into the reconstruction process. This is a challenging but promising research direction. The idea is to constrain the reconstruction not just by image data fidelity but also by physical plausibility. For example, one could include an energy term in the non-rigid tracking optimization that penalizes deviations from a physical model (like Hooke’s law for stretching, or an overall conservation of volume). If the object is known to be nearly incompressible (like water-filled tissue), the optimization can reject solutions where the volume changes drastically even if image error is small. Some works have begun exploring differentiable physics engines embedded in the reconstruction pipeline. These allow gradients of a physics simulation to flow into the reconstruction algorithm, so the system can tweak the shape to better satisfy physics (for instance, adjusting the reconstructed shape so that it would naturally deform into the next observed shape under its physical model). There have been proofs of concept where a template of the object with known material properties is used, and the non-rigid tracking ensures that the deformation of this template not only explains the images but also obeys Newtonian mechanics to some degree [40].

Another emerging area is the fusion of physics simulation with learning-based approaches to capture complex behavior that is hard to replicate. For example, simulating cloth with contact and fold dynamics is hard, but one can simulate offline with a high-fidelity physics engine and train a neural model on those results for use at runtime. The learned model can incorporate physics knowledge (via training data) and apply it quickly at runtime. There is also interest in expanding the scope of what physics these systems handle. Most current attempts, when they do include physics, focus on elasticity (spring-like behavior) and maybe simple damping. Yet real materials can have plastic deformation (permanent change of shape), tearing, self-collision with friction, etc. Handling these in real time is largely uncharted territory.

### 8.4. Computational Burden of Physics-Based Models

While incorporating physics into non-rigid reconstruction is desirable, it introduces a new challenge: heavy computational burden. Physics-based simulation of deformable objects (e.g., using finite element methods or mass-spring systems) is computationally expensive, especially when attempted in real time with high fidelity [40]. Solving the partial differential equations governing deformable motion or even computing forces and updates for many nodes of a mesh can quickly overwhelm real-time budgets, which are typically on the order of milliseconds per frame. Consequently, approaches that do include detailed physics tend to run much slower than real-time or require massive computing resources.

In the context of this gap, even though physics-based methods offer improved realism, they only form an emerging field in reconstruction and often target simple cases due to these performance issues [40]. High computational load not only slows down frame rates but also complicates the optimization: physics-based models have many variables and stiff equations that can lead to numerical difficulties. Therefore, there is a trade-off between physical accuracy and computational feasibility: systems either ignore physics to stay fast, or they handle physics with significant simplifications (linearized models, small deformation assumptions) to make the problem tractable. This gap is felt keenly in any attempt to scale to complex, high-resolution models or scenes—a full physics simulation for an object with thousands of vertices would not meet real-time constraints on today’s typical hardware without sacrificing detail. The computational burden gap is thus a barrier to using physics in practical, interactive applications. If not addressed, it means that even if we know how to model something physically, we might not be able to use that knowledge within a live system because it slows the system down too much.

To enable physics-based modeling in real time, research must focus on making physics simulation more efficient or approximating it in clever ways. One opportunity is to use model reduction techniques: simplifying the physical model to retain only the most important degrees of freedom. For example, instead of simulating every vertex of a high-resolution mesh, one could simulate a coarse deformation cage or a low-dimensional basis (from modal analysis or principal components) that captures the overall physical behavior. This reduced model can drive the full geometry by interpolation. Many deformable objects exhibit largely low-rank behavior (especially under moderate strain), so a small number of modes can approximate the deformation. By solving physics on this reduced basis, the computations become much faster, at the cost of some fine-grained accuracy. This approach has precedent in graphics (e.g., reduced physics models for real-time animation) and could be integrated into reconstruction: the system would enforce physics only on the coarse representation and rely on geometric refinement for fine details. Additional approximations reduce costs further. Under quasi-static assumptions, each frame is treated as an equilibrium state, valid when inertial effects are negligible. Alternatively, physics updates can run at a sub-rate of the camera frame rate with intermediate states obtained by interpolation, if appropriate—for instance, treating each frame as an equilibrium state if dynamic effects (inertia) are less important than elastic energy, or updating physics at a lower frequency than the camera frame rate and interpolating in between. These assumptions can cut down the number of computations and make a physically informed solution feasible within a limited time budget.

Another complementary avenue is to use machine learning as a substitute for physics computations. Instead of solving complex physics equations on the fly, a neural network or other regression model can be trained to predict the outcome of those equations. For example, given a current shape and external forces, a neural network could instantly predict the next deformed shape (effectively learning to integrate physics). This idea, often referred to as learning-based simulation or surrogate modeling, trades upfront offline training for extremely fast runtime inference. In the reconstruction context, one might train a network on simulation data of the object or category of interest, so that during real-time operation the network can enforce physical constraints by simply outputting a “corrected” shape that obeys those constraints. Some recent works demonstrate that neural networks can solve or assist with deformation PDEs orders of magnitude faster than classical solvers [40]. For instance, a network could learn to apply a correction to a geometrically reconstructed shape, adjusting it to satisfy elasticity or volume preservation in one step. These learned models can also be incremental, providing rapid convergence to the physical solution as an initial guess to a reduced number of physics solver iterations. Beyond networks, differentiable physics engines are becoming more mature; they allow gradient-based integration of simulation into learning frameworks. By using differentiable simulation, one can train models that incorporate physical knowledge, and at runtime those models essentially internalize part of the simulation, reducing how much computation is done explicitly.

Making use of parallel and high-performance computing remains an important opportunity. Physics simulations can be structured to exploit GPUs, as many are inherently parallel (computing forces on each element, for example). Modern libraries and game engines have shown real-time physics for graphics and games (like simplified cloth or soft body physics in interactive environments), suggesting that with optimized code and possibly specialized hardware (like FPGA or GPU kernels for physics), one can push closer to real-time even with substantial models. Combining all these strategies—model reduction, surrogate learning, and optimized computing—offers a path to embed physical realism in reconstruction without unbearable slowdowns. This will allow future systems to run at interactive rates while still capitalizing on the predictive and constraining power of physics models, effectively bridging the gap between high-fidelity simulation and real-time performance [40].

### 8.5. Emerging Challenges from Recent Work

The challenges discussed in this work, occlusion and viewpoint ambiguity, limited multi-object handling, inadequate physical modeling, and the computational burden of physics-based models are strongly echoed and elaborated in the most recent literature. A synthesis of the “Future work” sections across these papers suggests five transversal, still-unresolved issues.

Lack of unified, physics-aware benchmarks: As noted above, most methods are evaluated on bespoke or small-scale datasets tailored to specific tasks or sensing setups [42,45]. Digital-twin and world-model surveys explicitly identify the absence of standardized, physics-aware testbeds as a key obstacle to progress, and call for large-scale multi-modal benchmarks that combine modeling, rendering, and simulation of deformable objects under realistic interactions [45,46]. This observation reinforces the need for community efforts similar in spirit to the NRSfM animatronics benchmark but extended to volumetric dynamics and manipulation.

Accurate yet efficient models for complex materials and contact: While this review has shown that FEM, mass-spring, SPH, and PBD can all be adapted to real-time deformation to some extent, recent works emphasize that modeling elastoplasticity, heterogeneous tissues, and frictional contact with topological changes remains challenging under practical time budgets. EndoNeRF-based surgical twins, for instance, currently rely on relatively simple constitutive laws and standard solvers, and identify integration of more advanced schemes (e.g., XFEM, XMPM) as future work to better capture tumor resection and tissue cutting with acceptable latency [2,45]. Similarly, fast-simulation surveys and haptic-rendering reviews argue that despite promising progress in reduced-order and projective dynamics, the community still lacks robust methods that can guarantee stability and physical plausibility at haptic rates for highly detailed meshes and dense contact scenarios [21,49].

Robust multimodal perception under occlusion and transparency: Multiple surveys on deformable and fragile object manipulation highlight that severe self-occlusion, clutter, and transparent media remain major bottlenecks for vision-only pipelines, and argue for deeper integration of RGB-D, force, and proprioceptive/tactile sensing [50,51]. VIRDO, ViTaM-D, and the stretchable tactile glove confirm that distributed contact sensing and force-aware constraints improve reconstruction and tracking quality. However, generating large, high-quality visuo-tactile datasets remains laborious, and generalization beyond recorded objects and manipulation strategies is still limited [32,44]. Bridging the gap between fast, reactive tactile feedback and slower, deliberative visual perception is thus an open research direction, especially in safety-critical settings such as surgery and cooperative manipulation with humans [50].

Generalizable and safe control for various types of deformable objects: In general, learning-based methods that are used to control deformable objects work perfectly on tasks and material types that are used for training but do not generalize well to other geometries, stiffnesses, and fragilities [41,48]. The recent survey on deformable and fragile object manipulation identifies three architectural requirements: (i) fast, low-latency reflex loops (e.g., force or tactile); (ii) slower global optimization and planning layers; and (iii) explicit embedding of fragility and safety constraints in both objective functions [50]. At the same time, world-model surveys argue that such controllers should be grounded in 3D, physics-aware latent spaces to enable prediction and counterfactual reasoning under distribution shift, a capability that is still in its infancy for deformable bodies [46].

Real-time, deployable end-to-end systems: Finally, even when high-quality components exist for perception, simulation, and control, integrating them into end-to-end systems that meet demanding latency and robustness requirements on real robots remain largely unsolved. Digital-twin and world-model frameworks emphasize the need for model compression, adaptive level-of-detail, and hardware-aware scheduling (GPU/TPU acceleration, edge computing) to make real-time deformable-object reasoning feasible in robotics, AR/VR, and telepresence applications [45,46]. Similarly, differentiable-simulation frameworks such as gradSim and DPSI demonstrate the power of gradient-based identification and control from visual data, but their current computational costs and memory footprints still pose challenges for deployment in embedded or resource-constrained platforms [26,41].

Addressing these five issues will be central to the next generation of methods that seek to move beyond laboratory demonstrations and towards robust, physics-aware reconstruction and manipulation of flexible bodies in real-world environments.

### 8.6. Seminal Contributions Within the Survey Corpus

The five contributions highlighted in this section were identified through a structured selection process applied to the full corpus of 56 included works. Three criteria were applied conjointly. First, internal citation frequency works cited by five or more other papers within the corpus were flagged as candidates, indicating that they have directly shaped the research directions represented in this review. Second, technical representativeness: from the flagged candidates, works were retained if they introduced a methodological paradigm—rather than an incremental improvement—that is reflected in at least one of the four taxonomic categories of Figure 5 or one of the four systemic gaps identified in Section 8. Third, cross-domain influence: preference was given to works whose impact spans more than one of the application domains discussed in Section 6 (robotics, AR/VR, medical simulation), as these are more likely to represent foundational contributions to the field rather than to a specific subfield. Applying these three criteria to the corpus yielded seven candidate works; two were excluded because their primary contribution is a benchmark dataset rather than a methodological advance, and those are discussed separately in Section 7. The five-remaining works—covering dense monocular reconstruction, NRSfM evaluation, fast elastic simulation, differentiable system identification, and haptic rendering—collectively span the full methodological breadth of the review and are presented below. It is acknowledged that this selection process, while systematic, retains an element of judgment inherent to any qualitative synthesis, and that other reasonable selections from the corpus could be defended.

Within the corpus of 56 works considered in this review, a few contributions stand out as “seminal” in the sense that they are widely cited by other authors in the collection and have shaped subsequent research directions. We briefly highlight five such works and summarize how more recent papers build upon or challenge their findings.

#### 8.6.1. STAR on Dense Monocular Non-Rigid 3D Reconstruction

Tretschk et al. present a comprehensive STAR report on dense monocular reconstruction of deformable scenes, covering general objects, humans, faces, hands, and animals, and systematically discussing deformation models, optimization strategies, and open challenges. The report explicitly calls out the need for neural implicit representations, physics-aware reconstruction, and event-based sensing, and advocates for the integration of reconstruction and dynamics modeling [40]. These works directly instantiate the recommendations above: neural-field methods with hard constraints for PDE-governed fields [52], visuo-tactile implicit representations like VIRDO [33], NeRF-based surgical digital twins [2], and contact-centric deformation-learning schemes [25] all directly address the integration of implicit representations, physical constraints, and interaction that Tretschk et al. identify as crucial.

#### 8.6.2. NRSfM Benchmark and Evaluation

The benchmark and evaluation of NRSfM methods introduced by Jensen et al. constitute a second major milestone [42], providing, for the first time, a large-scale, multi-deformation dataset with accurate ground truth and a robust error metric. Their analysis demonstrates that many classical low-rank methods degrade significantly under perspective projection, occlusion, and non-ideal camera paths, and highlights the need for more expressive deformation priors and robust optimization [42]. Subsequent SfT and NRSfM works in this collection, including weakly supervised deep SfT trained directly on real videos [27], as well as hybrid physical–statistical approaches that combine particle-based dynamics with low-rank shape models [53], explicitly react to these findings by reducing dependence on fully labelled 3D data and by embedding stronger physical constraints into the reconstruction.

#### 8.6.3. Fast Simulation of Elastic Objects

Huang et al.’s survey on fast simulation of elastic objects synthesizes co-rotational FEM, model order reduction, modal warping, projective dynamics, and position-based methods into a coherent taxonomy and discusses their advantages and limitations for real-time applications [21]. This survey has become a standard reference for simulation-oriented work and directly underpins many later contributions that seek to combine physical realism with interactive performance. Contact-centric deformation learning adds high-frequency contact detail on top of subspace simulations via reduced models and neural approximations [25], NNWarp uses a neural network to regress non-linear deformations from a linear-elastic baseline [36], and NeuSpring builds a neural spring field on top of a spring-mass discretization to capture complex deformable behavior from videos [31]. Each of these methods can be interpreted as extending Huang et al.’s model-reduction paradigm with learned nonlinear corrections.

#### 8.6.4. Differentiable Simulation for System Identification and Control

GradSim introduces a unified differentiable multiphysics and rendering pipeline that enables gradient based identification of masses, friction coefficients, and elasticity parameters directly from video, as well as visuomotor policy learning from pixels [26]. This work has motivated a series of subsequent differentiable-simulation approaches for deformable objects DPSI adopts a similar philosophy for volumetric elastoplastic materials, estimating Young’s modulus, Poisson’s ratio, yield stress, and friction from a single short interaction using differentiable MPM and point-cloud-based loss functions [41].

NeuSpring likewise formulates reconstruction and simulation as a two-stage optimization problem over spring parameters and topology, and uses a canonical coordinate-based network to encode spring properties across frames [31]. NeRF-based surgical twins also build on this gradient-based paradigm, coupling differentiable volumetric rendering with FEM/MPM to enable data-driven soft-tissue simulation [2]. At the same time, these works highlight the computational and numerical challenges of scaling differentiable physics to high-resolution models and long horizons, issues that were already anticipated in gradSim’s discussion of computational cost and ill-posedness [26].

#### 8.6.5. Haptic Rendering of Deformable Objects

Xia’s et al. survey on new advances in haptic rendering provides a detailed overview of penalty-, constraint-, impulse-, and implicit-surface-based methods for rigid–rigid, rigid–deformable, and rigid–fluid interactions, and emphasizes the outstanding difficulties in achieving stability, high update rates, and physically realistic interaction for deformable objects and fluids [49,52]. The survey explicitly notes the limitations of mass-spring and linear FEM models at haptic rates and identifies reduced models combined with FEM as a promising direction [49]. This assessment is echoed by later visuo-tactile and haptic works in the corpus: VIRDO trades full FEM for an implicit SDF-based deformation field conditioned on visuo-tactile inputs [33], ViTaM-D uses FEM in an offline ZeMa simulator but relies on learned visuo-tactile constraints to enforce realistic contacts at reconstruction time [32], and the stretchable tactile glove system demonstrates how dense tactile sensing can be used to infer hand–object states without relying solely on high-frequency FEM simulation [44]. Neural-collision-detection methods further push this line of work by learning signed-distance approximations in reduced coordinates, significantly accelerating collision queries for deformable haptics [54].

These five seminal contributions, together with the broader body of work surveyed in this paper, collectively frame the current landscape of 3D reconstruction and real-time deformation of flexible bodies, and make clear that future advances will depend on a tight integration of robust sensing, physics-aware modeling, efficient numerical schemes, and carefully designed evaluation protocols [40,45].

The four challenges examined above—speed–accuracy trade-offs, occlusion fragility, limited multi-object handling, and the computational burden of physics—are not independent: they interact and reinforce one another in deployed systems.

The following Discussions section examines these interactions more deeply, analyzing how the choice of simulation paradigm (PBD vs. FEM), the role of model order reduction, and the integration of differentiable physics collectively shape the design space of future reconstruction–simulation pipelines.

## 9. Discussions

### 9.1. Integration of 3D Reconstruction and Real-Time Deformation

The integration of 3D reconstruction and real-time deformation modeling is a complex task that lies at the intersection of computer vision and physical simulation. It involves capturing the shape of a deforming object (3D reconstruction) and simultaneously computing or predicting its changes in shape (deformation) in a tightly coupled loop. Successfully combining these two aspects requires balancing accuracy, robustness, and speed, all while handling the sometimes-unpredictable dynamics of flexible bodies.

When integrating reconstruction and deformation, several challenges and considerations arise. First, achieving accurate and robust reconstruction is essential—any errors or noise in the captured geometry can negatively affect the deformation simulation. Traditional vision methods like stereo or SfM, as discussed, may falter under the rapid movements or appearance changes of deforming objects. Recent advancements in RGB-D sensing improve matters by providing direct depth measurements, but even these are limited by sensor range and can suffer from missing data on occluded or reflective surfaces [35]. Thus, a pipeline must be resilient to imperfect and partial data. This often means incorporating smoothing or regularization in reconstruction (to fill small holes or denoise) and possibly using predictive models to infer unseen parts.

Real-time performance is another critical consideration. The integrated system must process sensor data, update the 3D model, compute deformations, and possibly render results or feed information to a user/robot, all within a few milliseconds to maintain interactivity. This necessitates efficient algorithms and often hardware acceleration. Many systems employ parallel processing on GPUs to meet the demanding latency requirements of applications like VR or telerobotics.

For instance, non-rigid registration steps (aligning the current depth frame to the model) can be parallelized, and physics simulations can run on GPU as discussed. Even with such optimizations, balancing computational load is key: parts of the pipeline might be simplified to ensure the slowest stage does not bottleneck the whole system. For example, one might accept a slightly lower reconstruction resolution if it means the physics simulation can run faster, achieving a higher overall frame rate [35].

Flexible bodies introduce additional complexity due to their continuous shape change, which means the system must constantly re-fit the model to new sensor data. Unlike rigid SLAM (Simultaneous Localization and Mapping), where a single static model is incrementally built, here the model itself is deforming over time. Many methods use a deformable model representation such as a deformation graph or a mesh with embedded handle points that can move. Each incoming frame of data (depth map, point cloud, etc.) is used to update the node positions of this deformation graph to best explain the new observations.

This is essentially a non-rigid registration problem at each timestep, often solved by minimizing an energy that has a data term (bringing the model to the data) and a regularization term (like ARAP, to keep the deformation smooth and plausible). Ensuring this optimization converges quickly and reliably is challenging, especially when the object undergoes fast or complex motions. Some systems incorporate prediction: using the previously estimated motion, they extrapolate the model forward as a guess for the next frame, which can then be refined by aligning to the new data [55].

Hybrid methods that combine physics-based and data-driven components are increasingly employed to tackle integration challenges. A hybrid approach might use physics-based simulation to enforce the physical consistency of the model’s deformation while using data-driven inference to handle ambiguous or unobserved aspects. A physics-based model can reconstruct realistic deformations in occluded regions where visual data is absent, using material laws to infer the local state. A data-driven module can predict overall motion from past frames and use that prediction to initialize or constrain the physics simulation. Conversely, data-driven components can accelerate physics: a neural network might predict an initial guess of the deformation field, which the physics model then corrects as needed [56]. There are also hybrid formulations in the sense of algorithmic fusion: some methods integrate the reconstruction and deformation tasks into a single optimization problem, rather than treating one as input to the other. These formulations can ensure that the solution is globally optimal (the reconstructed shape is physically plausible, and the simulated deformation explains the sensor data) but are often complex to solve in real time.

Integrating 3D reconstruction with real-time deformation requires careful system design that addresses sensor limitations, computational constraints, and the need for physical realism. Practical solutions combine multiple strategies: multi-view or multi-sensor setups reduce occlusion; GPU-accelerated solvers improve throughput; precomputed models or learned priors provide stability; and adaptive computation focuses on processing regions with the highest deformation complexity; multi-view or multi-sensor setups are used to reduce occlusions, GPU-accelerated algorithms are used for speed, precomputed models or priors are used for stability, and adaptive methods that allocate more effort are used where and when it is needed (such as focusing computation on regions undergoing complex deformation). By leveraging the complementary strengths of vision and simulation, integrated systems aim to create a live digital replica of a deforming object or scene—one that not only looks geometrically correct but also behaves following physical laws.

To complement the qualitative analysis presented throughout this review, Table 9 provides a structured quantitative comparison of the principal method families discussed, aggregating performance figures reported across the 56 included works. Where individual papers report results on different hardware configurations or object categories, ranges are given rather than point estimates, and the corresponding representative references are cited. Metrics cover four dimensions identified as critical by the research questions of this review: reconstruction or inference speed (FPS), geometric accuracy (Chamfer Distance or mean surface error where available), robustness to occlusion (qualitative rating derived from the synthesis in Section 8.2), and indicative computational cost (GPU memory and processing class). It should be noted that direct numerical comparability across methods is limited by the absence of a unified benchmark, a gap explicitly identified in Section 8—this table should therefore be interpreted as an indicative synthesis rather than a definitive ranking.

FPS values reflect performance on desktop GPU hardware (NVIDIA RTX 3090 class or equivalent) as reported in the cited works; results on embedded or edge hardware are typically 3–5× lower. Chamfer Distance (CD) values are reported in millimeters and refer to reconstructions of objects in the 10–50 cm size range. Occlusion robustness ratings (Low/Medium/High) are derived qualitatively from the synthesis in Section 8.2 and Table 7. N/A indicates that the metric is not applicable or not reported for that method family.

### 9.2. Speed vs. Physical Fidelity Trade-Off

#### 9.2.1. Comparative Analysis: PBD Stability vs. FEM Material Accuracy

The choice between Position-Based Dynamics (PBD) and the Finite Element Method (FEM) represents a fundamental trade-off in the simulation of flexible bodies, particularly in the context of real-time 3D reconstruction. Within the corpus of 56 included works, PBD or its extended variant XPBD is used as the primary simulation backend in 8 of the 12 works classified as hybrid or application-specific pipelines targeting real-time visual feedback (Table 4, Appendix A). This preponderance reflects PBD’s unconditional numerical stability and low per-iteration cost rather than its physical accuracy and is consistent with its widespread adoption in commercial game engines and AR/VR frameworks [21,24]. FEM-based backends appear in works where quantitative material accuracy is required—primarily surgical simulation [2,20] and haptic rendering [49]—confirming that the choice between PBD and FEM is application-driven rather than reflecting a consensus on physical correctness.

#### 9.2.2. The Role of Model Order Reduction (MOR) as a Bridge

The primary obstacle to the widespread adoption of Finite Element Method (FEM) formulations in real-time flexible body reconstruction is their inherent computational complexity, which typically scales at O(n^3^) or O(n^2^) depending on the specific solver architecture. Model Order Reduction (MOR) serves as a critical technological bridge to overcome this bottleneck by leveraging the observation that a flexible body does not deform in an infinite variety of ways but rather follows predictable patterns. By performing modal analysis during a pre-processing phase, MOR identifies the most significant vibration modes or basis vectors that characterize an object’s likely deformations. This allows for drastic dimensionality reduction, where the high-dimensional FEM equations are projected into a reduced subspace. Consequently, instead of calculating the displacement of thousands of individual mesh vertices, the system operates within a manifold of significantly lower dimensionality, often reducing degrees of freedom from tens of thousands to a mere 20 or 50.

This mathematical approximation is transformative for interactivity, enabling FEM-based simulations to achieve the high update rates required for visual feedback (60+ FPS) and haptic rendering (1000 Hz) without sacrificing the physical integrity of the underlying material properties. The integration of MOR into the reconstruction–simulation pipeline facilitates the development of physics-aware digital twins that go beyond mere surface tracking. Because the reduced model remains grounded in FEM principles, the system can estimate internal stress distributions and enforce volume consistency in real time. Such capabilities are indispensable for high-fidelity applications, including advanced robotic manipulation of deformable objects and personalized medical training simulations, where both visual accuracy and physical realism are paramount.

### 9.3. Geometric vs. Physics-Aware Metrics

The integration of Contact-Intersection-over-Union (Contact-IoU) and stress-based objectives represents a significant paradigm shift in evaluation methodologies for physical simulations. Unlike the traditional Chamfer Distance (CD), which primarily quantifies spatial proximity, Contact-IoU focuses on the functional interface between interacting bodies. In robotic manipulation scenarios—such as a gripper handling a silicone organ—Contact-IoU provides a more rigorous assessment of the contact patch accuracy. This distinction is vital, as a reconstruction may achieve a deceptively low CD while failing to maintain a valid Contact-IoU, resulting in ghost contacts or unphysical slippage within the simulator. Furthermore, for specialized domains like medical modeling or structural analysis, the internal state of the material is as critical as its surface representation. Modern frameworks address this by incorporating stress–strain tensors directly into the loss function, ensuring that deformations are not merely visually plausible but are strictly governed by the material properties, such as Young’s Modulus, defined within the physics engine.

A central thesis of recent research is that minimal geometric error does not inherently guarantee a realistic or functional simulation. A reconstruction pipeline may achieve sub-millimeter precision in surface tracking while simultaneously yielding physically impossible results, such as self-collision or interpenetration. Because standard geometric metrics often fail to penalize mesh-to-mesh intersections, a model might perfectly align with a 3D scan of folded cloth while allowing the layers to pass through one another. In robotics, this inability to detect collisions renders a digital twin ineffective for reliable motion planning. Similarly, many flexible materials, such as human tissue or elastomers, are characterized by near-incompressibility. Without imposing differentiable physical constraints or specific Poisson ratios, neural network-based reconstructions artificially shrink or stretch the object to fit the visual data. Such violations of volume conservation laws prevent the model from accurately predicting how an object will react to external pressure, highlighting the need for physics-aware evaluation metrics.

### 9.4. The Role of Differentiable Physics

Building on the technical foundations presented in Section 5.5, this section examines how differentiable simulators address the four systemic gaps identified in this review, and what limitations remain.

#### 9.4.1. The Black-Box Limitation in Flexible Body Research

Early data-driven approaches (2014–2018), reviewed in Section 5.4, relied on standard CNNs and MLPs to predict vertex positions directly. While fast at inference, these models lack any internal representation of mass, stress, or constitutive laws. As a result, they interpolate between training frames rather than generalizing from physical principles, producing outputs that may be geometrically plausible but physically inconsistent. This limitation is particularly consequential in safety-critical contexts such as surgical simulation, where geometrically unverified deformation estimates carry non-trivial clinical risk.

#### 9.4.2. The Rise of Differentiable Simulators (DiffSim)

Differentiable simulators such as DiffTaichi [30] and gradSim [26] directly address two of the four gaps identified in Section 8: inadequate physical modeling and limited end-to-end sensing–simulation integration. By propagating gradients through the physics engine itself, these frameworks enable two capabilities unavailable to black-box networks.

First, parameter estimation from observation: rather than requiring hand-tuned material constants, a differentiable simulator can recover the exact Poisson ratio, stiffness, or friction coefficient of a specific object by backpropagating the visual reconstruction error through the physics engine [26,41]. This eliminates a major source of sim-to-real discrepancy identified in Section 8.3.

Second, multimodal fusion as physics-aware optimization: as highlighted in Section 9.5, differentiable simulators are naturally suited to reconciling heterogeneous sensor streams. The physics engine acts as a structured prior that constrains the solution space, ensuring that what the camera observes and what the tactile sensor measures are jointly consistent with a single physically plausible deformation state [26].

#### 9.4.3. A Balanced Assessment of Differentiable and Neural Methods

The preceding subsections have outlined the genuine methodological advances offered by differentiable simulators and learned deformation models. It is equally important, however, to situate these advances within an honest account of their current limitations, so that the research roadmap proposed in this review reflects realistic near-term constraints rather than an optimistic extrapolation.

Across the three dimensions of generalization, interpretability, and computational cost—identified by recent surveys as the primary barriers to deployment [40,47,50]—neural and differentiable methods currently occupy different positions on the capability frontier, but none has resolved all three simultaneously. Data-driven surrogates excel at inference speed but sacrifice generalization and interpretability. Differentiable simulators recover interpretable physical parameters and generalize within the identification regime, but at a computational cost that remains prohibitive for high-resolution or long-horizon problems. Physics-Informed Neural Networks (PINNs) offer a partial compromise by embedding physical constraints into the loss function, but their training convergence is sensitive to the relative weighting of data and physics terms, and their scalability to three-dimensional, contact-rich scenarios remains limited [40].

The sim-to-real gap—the systematic discrepancy between model predictions and real-world behavior arising from unmodeled physics, sensor noise, and distributional shift—affects all three paradigms, but manifests differently in each. For data-driven surrogates, it appears as catastrophic performance degradation on out-of-distribution inputs. For differentiable simulators, it manifests as parameter estimates that fit the training trajectory but fails to generalize to new interaction conditions. For hybrid frameworks, it arises at the interface between the learned and physics-based components, where the assumptions of each may be locally inconsistent.

These limitations do not diminish the importance of neural and differentiable methods as research directions; they define the specific technical problems that must be solved before these methods can underpin reliable, deployable systems. The research roadmap of Section 10 is formulated with these constraints explicitly in view.

### 9.5. Occlusion Fragility and Learned Spatiotemporal Priors

A persistent challenge in the reconstruction of deformable objects is the fragility of systems in the face of severe occlusions, where visual continuity is interrupted. Methods based on learned spatiotemporal prior-predictive models trained on temporal sequences can infer the geometry of occluded surfaces by extrapolating from observed states. These models additionally predict object dynamics from learned motion-deformation correlations, extending inference to unobserved surface regions.

However, this ability introduces the risk of generating visually plausible but physically inconsistent artifacts if the model is not constrained by mechanical laws. To mitigate this issue, recent research tends to integrate learned priors with differentiable physics solvers, ensuring that the “reconstructed” geometry in the unseen areas’ respects volume conservation and the elastic limits of the material.

Thus, the “hallucination” becomes an informed prediction, transforming occlusion from a critical error into a problem of probabilistic state estimation. Taken together, the discussions above converge on a unified picture: the most promising direction for this field is the tight coupling of physics-informed learning with multimodal sensing, underpinned by standardized evaluation. The following section synthesizes the main findings of this review into a set of conclusions and provides explicit answers to the four research questions that motivated it.

## 10. Conclusions

3D reconstruction and real-time deformation of flexible bodies represent a rapidly maturing yet still challenging frontier at the intersection of computer vision, physics simulation, and machine learning.

Sensing modalities have evolved from costly multi-camera rigs to consumer RGB-D devices and complementary tactile arrays.

Reconstruction pipelines have progressed from offline structure-from-motion to real-time neural implicit representations.

Deformation modeling has advanced from analytically derived FEM and mass-spring systems to data-driven surrogates and differentiable simulators.

Together, these developments have substantially improved the fidelity, speed, and accessibility of capturing and simulating flexible objects in motion. Despite this progress, four interconnected challenges continue to limit deployment in demanding real-world scenarios.

First, the speed–accuracy trade-off remains unresolved: physics-based simulations of sufficient fidelity routinely exceed real-time budgets, while faster alternatives such as PBD sacrifice material accuracy.

Second, occlusion and viewpoint ambiguity undermine reconstruction reliability whenever parts of a deformable object are hidden from all sensors, and current occlusion-aware methods provide only partial mitigation.

Third, most pipelines assume a single isolated object and break down when multiple deformable bodies interact, occlude each other, or exchange contact forces.

Fourth, reconstructions are frequently geometrically plausible but physically inconsistent—they fit the observed images without enforcing material laws, producing models that cannot reliably predict behavior under unseen forces or conditions.

Addressing these gaps will require progress along five concrete and interdependent directions, each associated with specific technical milestones and measurable success criteria.

Direction 1—Unified physics-aware benchmark: The field’s most pressing need is a community benchmark that jointly evaluates dense geometry, long-horizon dynamics, contact fidelity, and task performance across diverse flexible bodies. A minimally viable benchmark should satisfy five requirements identified in Section 7.6: multi-modal ground truth combining structured-light geometry with mechanically verified material parameters (Young’s modulus ±5% tolerance, Poisson’s ratio ±0.02) and calibrated tactile force distributions; a diversity of at least four object categories (cloth, soft tissue analogue, cable, foam elastomer) with at least ten geometric variants per category; a standardized evaluation protocol specifying five primary metrics—Chamfer Distance (CD), contact-IoU, penetration depth, task success rate, and PSNR/SSIM for rendering-based methods—computed on a reference GPU platform (NVIDIA RTX 4090 class or equivalent at time of publication); both controlled laboratory and unstructured real-world sequences to enable quantification of the synthetic-to-real gap; and full public availability under a CC-BY or equivalent license. A concrete short-term milestone would be the extension of the NRSfM Animatronics benchmark [42] with FEM-derived material ground truth and multi-view RGB-D streams, achievable within a two-year coordinated effort by three to four research groups.

Direction 2—Model order reduction for real-time FEM: Bridging the speed–accuracy gap requires reducing FEM complexity from O(n^3^) to O(k^3^) with k ≤ 50 retained modes while preserving physical consistency for the deformation regimes relevant to each application. Specific open challenges include: automated modal basis selection for heterogeneous materials where the dominant deformation modes are not known a priori; online basis adaptation when the object’s boundary conditions change during manipulation (e.g., a grasped tissue changes contact configuration); and error-bounded reduced models that provide certified upper bounds on the approximation error relative to the full FEM solution. A measurable milestone is a reduced FEM implementation achieving ≥60 FPS at ≤3 mm mean surface error for a 10^4^-element mesh of a soft tissue analogue on a single GPU, verified on a publicly available dataset.

Direction 3—Differentiable physics for material parameter identification: Differentiable simulators such as gradSim [26] and DPSI [41] demonstrate gradient-based identification of material constants from video, but face three unresolved challenges that limit deployment. First, ill-posedness: the inverse problem must be regularized by physically motivated priors (e.g., bounds on Young’s modulus derived from material class) to avoid convergence to physically implausible local minima. Second, contact differentiability: smooth contact approximations introduce systematic bias for dense contact scenarios; developing differentiable contact models with provable error bounds relative to the true discontinuous system is an open research problem. Third, scalability: current implementations are limited to meshes of approximately 10^4^ elements; achieving scalability to 10^5^–10^6^ elements requires checkpointing strategies and adjoint-based gradient computation that have not yet been demonstrated for flexible-body identification problems. A concrete milestone is parameter identification achieving Young’s modulus error ≤ 10% and Poisson’s ratio error ≤ 0.05 from a 5 s RGB-D sequence of a real elastomeric object, without manual initialization of material constants.

Direction 4—Occlusion-robust multimodal fusion: Closing the occlusion gap requires moving beyond single-modality reconstruction toward architectures that actively exploit complementary sensor streams. Three concrete advances are needed. First, learned spatiotemporal priors constrained by differentiable physics: the prior should predict occluded surface motion by propagating observed deformation through a reduced physical model, rather than purely through learned temporal correlations, ensuring that hallucinated geometry satisfies volume conservation and elastic limits. Second, uncertainty-aware reconstruction: methods should output calibrated confidence maps over surface regions, enabling downstream systems (robot planners, surgical guidance) to request additional observations when uncertainty exceeds application-specific thresholds. Third, standardized occlusion protocols: existing benchmarks do not systematically vary occlusion level, viewpoint count, or contact configuration; a benchmark extension defining five occlusion severity levels (0%, 20%, 40%, 60%, 80% of surface area occluded) would enable quantitative comparison of occlusion-robustness across methods for the first time.

Direction 5—End-to-end sensing–simulation integration: RQ4 identified end-to-end integration as the least mature component of the field. Current systems treat reconstruction and simulation as loosely coupled stages connected by a one-way data flow; closing this gap requires bidirectional coupling where simulation constraints regularize reconstruction and reconstruction outputs update simulation state in real time. Three architectural requirements must be satisfied simultaneously: latency below 33 ms (≥30 FPS) for the complete sensing–reconstruction–simulation loop on embedded hardware (NVIDIA Jetson AGX class or equivalent); physical consistency guarantees—specifically, non-penetration enforcement and volume conservation within ±1%—maintained throughout the loop without separate post-processing; and graceful degradation under sensor failure, where the loss of one modality (e.g., tactile arrays go offline) causes bounded rather than catastrophic performance degradation. A measurable milestone is a demonstrated end-to-end pipeline on a real robotic manipulation task achieving ≥25 FPS, ≤5 mm reconstruction error, and ≥80% task success rate on a standardized deformable object manipulation benchmark.

Returning to the four research questions that guided this review, the revised conclusions provide the following concrete answers.

RQ1 established that RGB-D cameras offer the most practical sensing modality for real-time flexible-body capture, achieving 30 Hz frame rates at sub-5 m range on consumer hardware, while LiDAR provides superior range and precision for large-scale or outdoor scenarios at higher cost, and tactile sensors provide irreplaceable sub-millimeter contact information that vision cannot recover under occlusion. The open challenge is automatic, online calibration and synchronization of heterogeneous sensor arrays without manual intervention.

RQ2 revealed that deformation-graph pipelines such as DynamicFusion [9] and neural implicit representations handle moderate deformations (<30% strain, no topological change) with reconstruction errors of 2–8 mm at real-time rates, but performance degrades by a factor of 2–5× under severe occlusion (>40% surface area hidden) and fails entirely under topology changes. No currently available method reliably handles both simultaneously.

RQ3 showed that no single deformation model dominates across all criteria: FEM achieves ≤1 mm material-consistent accuracy at 1–10 FPS for 10^3^–10^4^ element meshes; PBD achieves >30 FPS with 3–10 mm geometric accuracy but without material fidelity; and data-driven surrogates achieve 20–100+ FPS at 2–8 mm accuracy within their training distribution but degrade unpredictably outside it. The practical implication is that model selection must be application-driven, and hybrid architectures that combine PBD stability with FEM accuracy via model order reduction represent the most promising near-term path.

RQ4 identified end-to-end sensing–simulation integration as the field’s primary open challenge. No published system currently achieves simultaneous real-time performance (≥30 FPS), physical consistency (non-penetration, volume conservation), and occlusion robustness in a single deployed pipeline. Differentiable physics and unified learning frameworks represent the most promising technical path toward closing this gap, subject to the scalability and ill-posedness challenges identified in Direction 3 above.

The roadmap has been transformed from a list of aspirations into a verifiable research program, presented in Appendix B: each direction has a numerical success criterion, a realistic time horizon, and an explicit link to the gaps identified in Section 8.

## Figures and Tables

**Table 1:** Comparative overview of 3D reconstruction methods.

Method	Data Input	Strengths	Limitations
Stereo Vision	2+ images	Low hardware cost	Limited to textured surfaces
SfM	Unordered images	No camera calibration	Computationally intensive
RGB-D Sensors	Depth + RGB	Real-time capture	Limited range (5 m)
LiDAR	Laser point clouds	Long-range, high precision	Expensive hardware
Photogrammetry	2D images	High detail, flexibility	Lighting/occlusion sensitivity

**Table 2:** Key characteristics of dynamic 3D reconstruction algorithms.

Research Method	Mechanism	Paradigm	Key Characteristics & Advantages	Real-Time
DynamicFusion (2015)	6D Volumetric Warp Field [9]	Template-free (NRSfM-inspired)	First dense, template-free SLAM for dynamic scenes [9]	Yes
VolumeDeform (2016)	Depth + SIFT Features [11]	Template-free (NRSfM+RGB anchors)	Reduces drift and tracks tangential motion using color anchors [11]	Yes
KillingFusion (2017)	SDF Level-Set Evolution [10]	Template-free (NRSfM, correspondence free)	Correspondence-free; handles topological changes (merging/splitting) naturally [10]	Yes
Deep NRSfM (2021)	Hierarchical Sparse Coding [12]	NRSfM (unsupervised)	Unsupervised DNN; robust to occlusions and weak perspective projection [12]	No
Multi-body NRSfM (2024)	Group Dictionary Learning [13]	NRSfM (multi-body extension)	Reconstructs multiple interacting bodies from multi-view uncalibrated cameras [13]	No

**Table 3:** Comparison of neural radiance fields (NeRF) for dynamic scenes.

Feature	NeRF [14]	D-NeRF [15]	Li et al. [16]	Nerfies [17]
Primary Focus	Static scenes	Dynamic/Non-rigid	Space-Time synthesis	Casual selfies
Input	5D (Coords + View)	6D (Coords + View + Time)	Monocular video	Casual phone video
Key Innovation	Volumetric Rendering	Canonical mapping	Static + Dynamic MLPs	SE(3) fields & C2F reg.
Deformation	None	Translation vectors	Warping & Scene Flow	SE(3) rigid transforms

**Table 4:** Comparison of Position-Based Dynamics (PBD) vs. Finite Element Method (FEM).

Feature	Position-Based Dynamics (PBD)	Finite Element Method (FEM)
Core Principle	Operates directly on positions; uses geometric constraint projection to maintain shape.	Solves partial differential equations (PDEs) based on continuum mechanics (stress/strain).
Numerical Stability	Unconditionally Stable. It avoids numerical blow-up even with large time steps or extreme deformations.	Conditionally Stable. Often requires complex implicit solvers to remain stable under high-stress scenarios.
Physical Realism	Visual Approximation. Parameters like stiffness are phenomenological and do not map to real material constants.	High Fidelity. Accurately models Young’s modulus and Poisson’s ratio, essential for medical/bio-mechanical accuracy.
Computational Cost	Low. Very fast and easily parallelizable on GPUs, making it ideal for AR/VR and gaming.	High. Solving large matrices is traditionally too slow for 1000 Hz haptic or high-speed robotic feedback.

**Table 5:** Comparative analysis of deformation modeling approaches: accuracy, efficiency, and stability.

Feature	Traditional Black-Box Neural Networks	Differentiable Simulators (e.g., DiffTaichi, GradSim)
Core Mechanism	Learns a direct mapping from input (e.g., force) to output (e.g., mesh deformation) via hidden layers.	Embeds physical laws (FEM, PBD) as differentiable layers within a neural pipeline.
Physical Consistency	Low. May produce results that appear visually plausible yet violate volume conservation or gravity.	High. The output is inherently constrained by the underlying physical equations.
Data Efficiency	Low. Requires massive datasets (thousands of simulations/scans) to learn basic physical behaviors.	High. Can learn from very few samples because prior knowledge of physics is already built-in.
Generalization	Struggles with out-of-distribution scenarios (e.g., a much heavier object or different material).	Strong generalization; it calibrates physical parameters (friction, stiffness) rather than just mimicking motion.
Interpretability	Opaque. It is difficult to understand why a specific deformation occurred.	Transparent. Changes in output can be traced back to specific physical properties like Young’s Modulus.

**Table 6:** Existing datasets for evaluating reconstruction and deformation methods.

Dataset/Benchmark	Primary Evaluation Target	Sensing Modality	Ground-Truth Type	Physics Parameters Available	Contact/Multi-Object Support	Availability	Key Reference
NRSfM Animatronics	NRSfM geometric tracking	Structured-light scanner	Dense 3D surface per frame	No	No	Public	[42]
ACID/PlushSim	Implicit visual dynamics, goal-conditioned manipulation	RGB video (synthetic)	Action trajectories, volumetric shape	Partial (elasticity class)	Limited (single object)	Public	[43]
NeuSpring	Reconstruction + spring-mass simulation	RGB-D video	3D mesh sequence	Learned spring parameters	No	Upon request	[31]
HOT (ViTaM-D)	Hand–object deformable interaction	Multi-view RGB-D + tactile arrays	Contact patches, penetration depth, FEM mesh	Yes (FEM-based)	Yes (hand + object)	Upon request	[32]
Stretchable Tactile Glove corpus	Contact-rich manipulation of elastic objects	Multi-view RGB + 1152-ch tactile	Contact localization, force distribution	Partial	Yes (rigid + elastic)	Upon request	[44]
DexYCB	Hand–object manipulation (rigid/articulated)	Multi-view RGB-D	6-DoF object pose, hand keypoints	No	No (rigid objects only)	Public	[32]
SoftGym	Deep RL for deformable manipulation	Synthetic RGB (FleX engine)	Particle positions, task success	No (PBD constraints only)	Limited	Public	[24]
EndoNeRF/FastEndoNeRF	Soft tissue reconstruction (surgical)	Stereo endoscopic video	Depth maps, PSNR/SSIM	No	No	Partial	[2]
gradSim evaluation sequences	System identification from video	RGB video (synthetic + real)	Material parameters (E, ν, friction)	Yes	Limited	Partial	[26]
DPSI evaluation sequences	Elastoplastic parameter identification	RGB-D + point clouds	Young’s modulus, yield stress, friction	Yes	Limited	Upon request	[41]

**Table 8:** Taxonomy and computational trade-offs of deformations/reconstruction frameworks.

Method Category	Computational Cost (FPS/Complexity)	Robustness to Occlusion	Physical Consistency	Primary Use Case
Geometric Based	High FPS (30–60+). Efficient for surface-only tracking.	Low. Rely heavily on visual line-of-sight.	None. Only visual plausibility; no mass or energy conservation.	Real-time AR filters, simple surface tracking.
Physics-Based	Variable. PBD is fast (30+ FPS); FEM is slow (<10 FPS).	Medium. Can predict motion briefly via momentum.	High. Built on laws of mechanics (stress/strain).	Medical surgery training, high-fidelity cloth/tissue simulation.
Data-Driven	High Inference Speed: (20–50 FPS) FPS for mesh/point-cloud models [29,37]; >100 FPS (sub-millisecond latency) for compact latent-space networks [3]	Medium to High. Can infer occluded geometry from learned priors.	Low to Medium. Depends on training data diversity.	Rapid prototyping, motion prediction from sparse sensors.
Hybrid Frameworks	Moderate (15–30 FPS). Balanced for real-time loops.	High. Combines visual data with physical priors to fill gaps.	High. Constrained by differentiable physics layers.	Autonomous robotic manipulation, complex contact-rich scenes.

**Table 9:** Quantitative comparison of method families.

Method Family	Representative Methods	Inference Speed (FPS)	Geometric Accuracy (CD/Surface Error)	Occlusion Robustness	Computational Cost	Key References
Geometric-Based	DynamicFusion, VolumeDeform, KillingFusion	25–30 FPS (real-time)	2–8 mm (depth-dependent)	Low—relies on continuous visual line-of-sight	Medium GPU (4–8 GB VRAM)	[7,8,9]
FEM-Based (full-order)	Co-rotational FEM, SPH	1–10 FPS (sub-real-time)	<1 mm (high fidelity)	Medium—physics extrapolation for short gaps	High GPU/CPU (16+ GB)	[21,22,46]
FEM + Model Order Reduction	Modal FEM, Projective Dynamics	30–60 FPS	1–3 mm	Medium	Medium GPU (8–12 GB)	[46,52]
Position-Based Dynamics (PBD)	XPBD, SoftGym	>30 FPS (up to 120 FPS)	3–10 mm (material approximation)	Medium—momentum-based prediction	Low–Medium GPU (4–8 GB)	[28,46]
Neural/Implicit (NeRF-based)	D-NeRF, Nerfies, EndoNeRF	0.1–5 FPS (optimization-heavy)	<2 mm (photometric)	Low–Medium—limited to observed views	High GPU (16–24 GB)	[13,15,30]
Data-Driven Surrogates (GNN)	MeshGraphNets, GNS	20–50 FPS	2–6 mm (task-dependent)	Medium–High—learned priors infer occluded regions	High training/Low inference GPU	[34,35,42]
Compact Latent-Space Networks	Neural cloth (Lee et al.)	>100 FPS (sub-ms latency)	4–8 mm (wrinkle-level detail)	Medium—limited generalization	Low inference GPU (4 GB)	[23]
Differentiable Simulators	DiffTaichi, gradSim, DPSI	5–30 FPS (identification mode)	<1 mm (parameter-calibrated)	High—physics-constrained inference	Very High (32+ GB, multi-GPU)	[25,31,43]
Hybrid Frameworks	Visuo-tactile pipelines, ViTaM-D	15–30 FPS	1–4 mm	High—combines visual and tactile constraints	High GPU + tactile hardware	[39,40,51]

## Data Availability

The original contributions presented in this study are included in the article. Further inquiries can be directed to the corresponding author.

## Appendix A

**Table A1:** Summary of physics-based simulation methods, applications, and key characteristics.

Work (Author, Year)	Method	Application	Key Characteristics
Finite Element Method (FEM) & Analytical/Mechanical Models			
Agudo, 2016	Sequential EKF-FEM	Dense non-rigid reconstruction	Simultaneous estimation of camera motion and deformable shape in real time.
Luengo-Sanchez, 2025	Analytic Shape-from-Template (SfT)	Reconstruction via Partial Differential Equations (PDE)	Study on isometric, conformal, and equiareal deformation models.
Marinkovic, 2019	Co-rotational Finite Element (FEM)	VR-based surgery simulators	Analysis of real-time FEM simulation possibilities on conventional hardware.
Miller, 2017	Simscape Multibody (multibody modeling)	Crank-slider mechanisms, beams	Capturing the effects of small elastic deformations in mechanical systems.
Zhu, 2025	Analytical Models (FEM, Mass-spring)	Manipulation of fragile objects	Compromise between physical fidelity and computational speed in robotic control.
Material Point Method (MPM) & Differentiable Programming			
Du, 2023	Differentiable Physics & Deep Fluids	Robotic locomotion and fluid flows	Use of mechanics-informed neural networks for constitutive modeling.
Hu, 2019	Differentiable MLS-MPM (ChainQueen)	Soft robotics and solid–fluid coupling	Real-time simulator based on continuum mechanics with back-propagation.
Hu, 2020	DiffTaichi (Differentiable Programming)	Video games and robotic control	High-performance language for automatic derivatives in complex physical simulations.
Wang, 2024	Cd-MPM & AnisoMPM	Dynamic fracture animation	Modeling of anisotropic damage mechanics for complex materials.
Yang, 2025	Differentiable Depth (Real2Sim)	Soft body simulation calibration	Online estimation of model parameters for linear objects.
Neural Networks, Machine Learning & Implicit Representations			
Blackseth, 2022	Hybrid Analysis and Modeling (CoSTA)	2D thermal diffusion	Combines physical models (PDE) with neural networks to correct unknown source terms.
Huang, 2023	Graph Neural Networks (GNN)	Robotic manipulation of deformable objects	Evaluation of grasping quality through simulation-based metrics.
Koo, 2025	NeRF and 3D Gaussian Splatting (3DGS)	Digital Twins (DT) for deformable objects	Integration of modeling, rendering, and ML-based simulation.
Kularkar, 2023	GAN (ORGAN) and Tomography	Archaeology and medicine	Repairing archaeological objects and VR reconstruction.
Lee, 2024	Pose Space Deformation (PSD)	Dynamics of clothing items	Unsupervised learning of deformations based on pose sequences.
Park, 2020	DeepSDF & Implicit Representations	Continuous shape representation	Learning signed distance functions for 3D reconstruction.
Pfaff, 2021	Graph Networks for Mesh-Based Simulation	Tearing thin sheets and turbulent flows	Learning mesh-based simulations through physics-informed models.
Pumarola, 2020	D-NeRF (Neural Radiance Fields)	Reconstruction in function space	Learning implicit 3D representations without direct supervision.
Sanchez, 2020	Learnable Physics Engines	Fluid simulation and complex interactions	Using graph networks to learn particle dynamics from data.
Shen, 2023	Multi-task Neural Networks & StarGAN	Visual navigation and image synthesis	Learning generative vector representations for 3D objects.
Zhong, 2023	Factor Fields & Constrained Optimization	Deep Appearance Modeling	Unified framework for neural fields with optimal linear constraints.
Position-Based Dynamics & Particle Methods (PBD/SPH/PBF)			
Feida Gu, 2023	Position-Based Dynamics (PBD)	Surgical simulation and cloth-like objects	Comparative analysis between visual fidelity and physical interpretation.
Lin, 2020	Position Based Fluids/Dynamics	Real-time particle physics	Stable methods for simulating fluids and soft bodies.
Liu, 2015	Smoothed Particle Hydrodynamics (SPH)	Virtual surgery and diesel maintenance	Use of meshfree methods for real-time simulations.
Non-Rigid Structure from Motion (NRSfM) & Subspace Models			
Jensen, 2021	NRSfM Benchmark (physical vs. statistical)	Reconstruction through bending, stretching, tearing	Taxonomy of Non-rigid Structure from Motion methods.
Romero, 2022	Subspace Dynamics & Projective Dynamics	3D human mesh registration	Model order reduction by learning deformation maps.
Salzmann, (n.d.)	Linear Subspace Models (NRSfM)	Markerless garment capture	Use of smoothness and temporal consistency constraints in monocular reconstruction.
Tretschk, 2023	Dense Monocular NRSfM	Rendering deformed distance fields	Current state of the art in dense monocular 3D reconstruction.
3D Reconstruction, Sensors & Optical Techniques			
Du, 2009	Measuring Devices (GPS, Total Stations)	Forensic investigations	Evaluation of traditional methods’ efficiency vs. laser scanning.
Inman, 2016	Shading-based refinement	Online non-rigid 3D reconstruction	Real-time refinement of models obtained from RGB-D data.
Newcombe, 2015	DynamicFusion (volumetric warp field)	Non-rigid reconstruction from depth	First system capable of online reconstruction without a pre-scanned model.
Slavcheva, 2017	KillingFusion (SDF evolution)	3D reconstruction from RGB-D stream	Isometric flow-based approach without explicit correspondences.
Tachella, 2019	Sequential Single-Photon Lidar	Long-range depth profiling	Efficient computational imaging by detecting scattered photons.
Wu, 2022	TLS Scientometric Analysis	AEC (Construction) Industry	Trends evaluation in deformation monitoring and BIM modeling.
Zhou, 2024	Active/Passive 3D Reconstruction	Autonomous driving and VR	Comparative study between explicit and implicit geometric representations.
Zollhofer, 2014	Online Template Acquisition	Non-rigid reconstruction at 30 Hz	Use of GPU-based Gauss–Newton optimizer for detail integration.
Syntheses, Surveys & Methodological Reviews			
Huang, 2019	Position-based and co-rotational dynamics	Rapid simulation of elastic objects	Survey of methods for reducing computational complexity.
Jatavallabhula, 2021	Differentiable Rendering	Scene dynamics perception	Review on integrating automatic differentiation in vision and robotics.
Xie, 2025	MPM, PBD, and FEM Unified Survey	Inverse rendering and object manipulation	Multimodal generative models for real-world simulation.
Yunus, 2024	Hybrid Scene Representation	Novel View Synthesis (NVS)	Review of controllable non-rigid reconstruction methods.
Other Methods (Haptics, Mass-Spring Systems)			
Kenwright, 2011	Hexahedral mass-spring systems	Interactive games and medical simulations	Focus on artistic visual results with low computational cost.
Xia, 2018	Haptic Rendering (spring-damper)	Rigid–deformable interaction in medicine	Real-time contact force simulation for endoscopic procedures.

## Appendix B

**Table A2:** Research roadmap: open challenges, required advances, and measurable milestones.

Priority	Open Challenge	Required Technical Advance	Measurable Milestone	Target Horizon
1	Absence of unified benchmark	Multi-modal dataset with mechanical ground truth, 5-metric protocol, public release	Benchmark covering 4 object categories, 10 variants each, CC-BY license	2–3 years
2	FEM speed–accuracy gap	Online MOR with k ≤ 50 modes, online basis adaptation, certified error bounds	≥60 FPS, ≤3 mm error, 10^4^-element mesh, single GPU	2–4 years
3	Differentiable physics scalability	Adjoint gradients, differentiable contact with error bounds, regularized identification	E error ≤ 10%, ν error ≤ 0.05 from 5 s RGB-D sequence, no manual init	3–5 years
4	Occlusion robustness	Physics-constrained spatiotemporal priors, calibrated uncertainty maps	Standardized 5-level occlusion protocol; robust at ≤60% occlusion	2–4 years
5	End-to-end integration	Bidirectional reconstruction–simulation coupling, graceful sensor degradation	≥25 FPS, ≤5 mm error, ≥80% task success on standard benchmark, embedded hardware	4–6 years
